# Risk factors and risk prediction models for colorectal cancer metastasis and recurrence: an umbrella review of systematic reviews and meta-analyses of observational studies

**DOI:** 10.1186/s12916-020-01618-6

**Published:** 2020-06-26

**Authors:** Wei Xu, Yazhou He, Yuming Wang, Xue Li, Jane Young, John P. A. Ioannidis, Malcolm G. Dunlop, Evropi Theodoratou

**Affiliations:** 1 Centre for Global Health, Usher Institute, University of Edinburgh, Edinburgh, EH8 9AG UK; 2 Henan Provincial People’s Hospital, Henan, 450003 People’s Republic of China; 3 Sydney School of Public Health, University of Sydney, Sydney, NSW 2006 Australia; 4 Department of Medicine, School of Medicine, Stanford University, Stanford, CA 94305 USA; 5 Department of Epidemiology and Population Health, School of Medicine, Stanford University, Stanford, CA 94305 USA; 6 Department of Biomedical Data Science, School of Medicine, Stanford University, Stanford, CA 94305 USA; 7 Meta-Research Innovation Center at Stanford (METRICS), Stanford University, Stanford, CA 94305 USA; 8 Department of Statistics, School of Humanities and Sciences, Stanford University, Stanford, CA 94305 USA; 9 Colon Cancer Genetics Group, Medical Research Council Human Genetics Unit, Medical Research Council Institute of Genetics & Molecular Medicine, University of Edinburgh, Edinburgh, EH4 2XU UK; 10 Edinburgh Cancer Research Centre, Institute of Genetics and Molecular Medicine, University of Edinburgh, Edinburgh, EH4 2XU UK

**Keywords:** Colorectal cancer, Metastasis, Recurrence, Risk factors, Risk prediction models, Umbrella review

## Abstract

**Background:**

There is a clear need for systematic appraisal of models/factors predicting colorectal cancer (CRC) metastasis and recurrence because clinical decisions about adjuvant treatment are taken on the basis of such variables.

**Methods:**

We conducted an umbrella review of all systematic reviews of observational studies (with/without meta-analysis) that evaluated risk factors of CRC metastasis and recurrence. We also generated an updated synthesis of risk prediction models for CRC metastasis and recurrence. We cross-assessed individual risk factors and risk prediction models.

**Results:**

Thirty-four risk factors for CRC metastasis and 17 for recurrence were investigated. Twelve of 34 and 4/17 risk factors with *p* < 0.05 were estimated to change the odds of the outcome at least 3-fold. Only one risk factor (*vascular invasion for lymph node metastasis [LNM] in pT1 CRC*) presented convincing evidence. We identified 24 CRC risk prediction models. Across 12 metastasis models, six out of 27 unique predictors were assessed in the umbrella review and four of them changed the odds of the outcome at least 3-fold. Across 12 recurrence models, five out of 25 unique predictors were assessed in the umbrella review and only one changed the odds of the outcome at least 3-fold.

**Conclusions:**

This study provides an in-depth evaluation and cross-assessment of 51 risk factors and 24 prediction models. Our findings suggest that a minority of influential risk factors are employed in prediction models, which indicates the need for a more rigorous and systematic model construction process following evidence-based methods.

## Background

Around 20–25% of patients with colorectal cancer (CRC) present with metastasis at initial diagnosis, while patients who are apparently cancer-free on investigation at diagnosis subsequently develop locoregional recurrence (18%), distant (78%) recurrence, or both (4%) [1]. Metastasis occurs when cancer cells from the original tumor are able to proliferate in local, regional, or distant tissues; lymph nodes; or organs via lymphatic, blood, or even trans-coelomic spread [2]. CRC recurrence is defined as local, regional, and distant metastatic recurrence after a disease-free period [3]. Local recurrence refers to CRC relapse that occurs at the site of original surgical resection [4], while regional recurrence occurs at draining lymph nodes and/or lateral pelvic lymph nodes [3]. Distant metastatic recurrence involves the liver (accounts for 40–50% of metastases), the lung (accounts for 10–20% of metastases), the peritoneum, the ovaries, the adrenal glands, the bone, and the brain [1, 5]. It is estimated that 5-year survival rates are around 90%, 70%, and 10% for CRC localized, regional, and distant metastatic stages [6].

Validating individual risk factors and even more so multivariable prediction models of multiple risk factors for local, regional, or distant metastasis and recurrence is crucially important as these could guide management of the primary tumor and provide prognostic information for patients and their cancer clinicians. Prediction models may be more successful if they consider the most informative factors. This knowledge may eventually prove useful in managing CRC treatment with better-informed patient choices. Understanding the underlying validity and predictive performance of risk factors for locoregional recurrence is particularly relevant, given progressive moves towards organ-preserving approaches such as endoscopic resection (EMR), trans-anal microscopic surgery (TEMS), and neo-adjuvant chemoradiotherapy for rectal cancer [1], since organ preservation may be at the expense of elevated recurrence rates. The corollary also applies since the risk-benefit ratio of extensive locoregional surgery and/or radiotherapy may be detrimentally impacted by future distant metastases.

A number of systematic reviews (with/without meta-analyses) have investigated existing risk factors for CRC metastasis and recurrence [7–10]. However, there is a need for a comprehensive evaluation of the available epidemiological evidence. Here, we conducted an umbrella review to identify and evaluate associations between risk factors and risk of CRC metastasis and recurrence. We also systematically collected and evaluated predictive models on CRC prognostic outcomes. We then conducted a comparative cross-assessment between the identified risk factors and the predictors employed in risk prediction models to examine to what extent predictive models include the most influential factors.

## Methods

### Protocol

The study protocol was developed in accordance with the reporting guidance in the Preferred Reporting Items for Systematic Reviews and Meta-Analyses Protocols (PRISMA-P) statement [11].

### Umbrella review of systematic reviews (with/without meta-analyses) of risk factors

#### Literature search and eligibility criteria

A systematic search was performed in PubMed, Cochrane Library (Wiley), Web of Science (Thomson Reuters), and EMBASE (Ovid) from inception to 7 October 2019, to identify systematic reviews of observational studies with or without a meta-analysis that evaluated the associations between risk factors and risk of metastasis and recurrence in CRC (Additional file 1: Table S1). We further hand-searched reference lists of the retrieved eligible publications to identify additional relevant studies. All identified publications went through a parallel review of the title, abstract, and full text (performed by WX and YM independently) based on pre-defined inclusion and exclusion criteria following “PICOS.” In particular, we included human participants from observational studies with no restriction to settings. Conversely, animal, in vitro, and in vivo experiments were excluded. For study outcomes, we included CRC metastasis (local, regional, or distant metastasis in tissues, lymph nodes, or organs at diagnosis) and CRC recurrence (local, regional, or distant metastatic recurrence in tissues, lymph nodes, or organs after a disease-free period). For study design, we included systematic reviews of observational studies with or without meta-analysis. Conversely, literature reviews, individual observational studies, systematic reviews, and meta-analyses that investigated the evidence on the efficacy of pharmaceutical drugs and therapeutic procedures were excluded. We included publications in peer-reviewed journals, and therefore, gray literature, comments, conference abstracts, and interviews were excluded.

#### Data extraction

Data were extracted by one investigator (WX) and checked by a second investigator (YH). For each included meta-analysis, the following items were extracted: study citation details, number of studies included, study design, study population, number of events and size of total population, risk factors, outcomes examined, reported summary meta-analytic estimates (e.g., risk ratio [RR], odds ratio [OR], hazard ratio [HR], the corresponding 95% confidence interval [95% CI], *p* value, and heterogeneity measures), instrument applied for quality and risk of bias assessment of component studies, and quality assessment result. The following items were further extracted from the individual component studies: study citation details, study design, study population, risk factors, outcomes examined, number of events and size of total population in exposed and unexposed groups, effect size, and 95% CI.

#### Evidence synthesis and evaluation

First, when two or more meta-analyses examining associations between the same risk factor and the same outcome were identified, the most recent meta-analysis of prospective cohort studies with the largest event number was prioritized and retained for further analysis. We also compared whether the results reported in overlapping meta-analyses were concordant in terms of direction, statistical significance, and association magnitude.

Second, we estimated the following metrics for each unique meta-analysis: (1) The summary effect size along with 95% CI was estimated based on random-effects models (DerSimonian and Laird (DL)) when the number of component studies was five or more and the Hartung-Knapp-Sidik-Jonkman (HKSJ) when the number of component studies was less than five [12, 13]. (2) Heterogeneity was assessed by the *I*^2^ statistic [14]. (3) The 95% prediction interval was estimated. (4) The small study effect was estimated by Egger’s regression asymmetry test [15]. (5) Excess significance was assessed by a chi-square test [16]. Based on these metrics and by applying a set of pre-defined criteria (Additional file 1: Table S8), we evaluated the credibility of the evidence for each risk factor and categorized the evidence as convincing, highly suggestive, suggestive, or weak [17, 18].

Lastly, for all meta-analyses that statistically represented at least 3-fold changes in the odds of the outcome, we evaluated the methodological quality and risk of bias based on the Assessment of Multiple Systematic Reviews 2.0 (AMSTAR 2.0) checklist [19]. We used an odds ratio of 3.0 as a threshold for what is a substantially large effect. There is no consensus on what an optimal threshold might be, but values between 2 and 5 are proposed typically [20].

#### Sensitivity analysis

We re-ran all meta-analyses by evaluating the outcome definitions of each individual component study reclassifying the outcomes to (i) CRC metastasis at presentation, (ii) CRC local recurrence after a disease-free period, and (iii) CRC distant recurrence after a disease-free period.

### Comparative cross-assessment of risk factors and risk prediction models

We performed a comparative cross-assessment between risk factors evaluated in the umbrella review and risk predictors included in existing prediction models. A recently published systematic review conducted by our team [21] investigated a total number of 15 prediction models for prediction of metastasis and recurrence in CRC patients with surgical resection (metastasis: *N* = 6; recurrence: *N* = 9). We updated the original search to identify studies developing and/or validating risk prediction models to predict metastasis and recurrence in all CRCs, with no restriction on whether the tumor was resected. We performed a systematic search in PubMed from inception to 7 October 2019 to identify eligible studies. We extracted data relevant to study design, study population, prediction outcome, prediction time horizon, predictors, model performance, and model presentation from each included study. We created a catalog of all variables that had been included across CRC metastasis prognostic models and separately across CRC recurrence prognostic models (presented in the same order as in the respective tables). We then assessed whether the included risk predictors were evaluated or not in the umbrella review described above. If yes, we also recorded the magnitude of the summary relative risk (typically odds ratio) and noted how many of those represented at least 3-fold changes in the odds of the outcome and how many had convincing or highly suggestive evidence in our assessment.

All statistical analyses were conducted in Stata, version 14.0 (StataCorp), and R, version 3.3.0 (R Foundation for Statistical Computing).

## Results

### Literature review

**Fig. 1:**
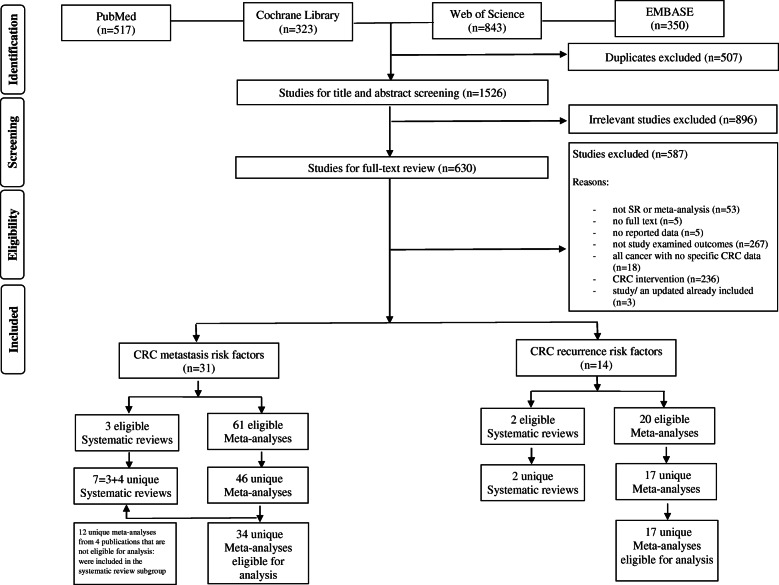
Flow chart summarizing the study identification and selection

A total of 2033 publications were retrieved from the systematic search in four databases. Eventually, 43 publications met all inclusion criteria (Fig. 1, Additional file 1: Table S2) and that included 9 systematic reviews (metastasis: *N* = 7; recurrence: *N* = 2) and 81 meta-analyses (metastasis: *N* = 61; recurrence: *N* = 20; Additional file 1: Table S3 and Table S4) of observational studies. A total of 18 overlapping meta-analyses that examined associations between the same risk factor and the same outcome were identified (Additional file 1: Table S5). The most recent meta-analysis with the largest event number was prioritized. Within the remaining 63 unique meta-analyses, 12 meta-analyses from four publications did not report detailed OR, RR, or HR in forest plots. Finally, 51 unique meta-analyses were retained for analysis, which reported 34 unique risk factors for CRC metastasis and 17 risk factors for recurrence (Additional file 1: Table S6 and Table S7).

### Meta-analyses of risk factors for CRC metastasis

Overall, 61 eligible meta-analyses of observational studies investigating risk factors for CRC metastasis were identified (Additional file 1: Table S3). More than one meta-analysis was conducted for seven risk factors (Additional file 1: Table S5). The direction of the summary effect size and the presence of nominal statistical significance (*p* < 0.05) of the reported associations in overlapping meta-analyses were concordant for six (86%) risk factors (Additional file 1: Table S5).

A total of 34 unique meta-analyses with available data were retained for further analysis (Additional file 1: Table S6). The median number of included component studies was five (range 2–41), the median number of the total population was 983 (range 76–10,128), and the median number of events was 138 (range 16–1808). The meta-analyses reported a wide range of risk factors (Additional file 1: Table S6): 17 histopathological risk factors (50%), 13 biomarkers (38%), three genetic risk factors (9%), and one demographic risk factor (3%). Overall, 21 (62%) of 34 unique meta-analyses reported effect sizes at *p* < 0.05 (Table 1). Based on the pre-defined credibility criteria, only one (3%) histopathological risk factor (*vascular invasion for LNM in pT1 CRC*) presented convincing evidence (see Additional file 1: Table S9 for the credibility assessment of all identified risk factors). Furthermore, 12 of 21 probed risk factors with *p* < 0.05 had an effect size suggesting ≥ 3-fold change in the odds of the outcome, while this was also seen for the point estimates in four of 13 probed risk factors where the meta-analysis had *p* ≥ 0.05 (Table 1).

**Table 1:** Summary of evidence credibility assessment of 34 unique meta-analyses of observational studies investigating the associations between risk factors and CRC metastasis

Population	Outcome	Risk factor	Risk factor prevalence	Effect size (95% CI)	Evidence classification
**Histopathological risk factor**					
pT1 CRC	Lymph node metastasis in pT1 CRC	Vascular invasion	330/1731 = 19%	2.73 (1.98–3.78)	Convincing
pT1 CRC	Lymph node metastasis in pT1 CRC	Lymphatic invasion	906/3347 = 27%	6.78 (5.29–8.69)	Highly suggestive
pT1 CRC	Lymph node metastasis in pT1 CRC	Tumor budding	2401/10,128 = 24%	6.39 (5.23–7.80)	Highly suggestive
CRC	Lymph node metastasis in CRC	Tumor budding	1955/6739 = 29%	4.96 (3.97–6.19)	Highly suggestive
Rectal cancer	Lymph node metastasis in rectal cancer	Tumor size > 1 cm	203/348 = 58%	6.76 (3.25–14.04)	Highly suggestive
pT1 CRC	Lymph node metastasis in pT1 CRC	Lymphovascular invasion	340/1695 = 20%	4.81 (3.14–7.36)	Suggestive
pT1 CRC	Lymph node metastasis in pT1 CRC patients who underwent additional surgeries after an endoscopic resection	Lymphovascular invasion	91/313 = 29%	5.29 (2.34–11.98)	Suggestive
pT1 CRC	Lymph node metastasis in pT1 CRC	Poor differentiation	94/2722 = 4%	5.61 (2.90–10.83)	Suggestive
Rectal cancer	Lymph node metastasis in rectal cancer	Muscularis properia invasion	122/322 = 38%	5.08 (2.32–11.11)	Suggestive
pT1 CRC	Lymph node metastasis in pT1 CRC	Submucosal invasion ≥ 1 mm	2389/2922 = 82%	2.95 (1.39–6.27)	Weak
Small rectal NETs	Lymph node metastasis in small rectal NETs treated by local excision	Lymphovascular invasion	104/517 = 20%	5.02 (1.16–21.72)	Weak
Rectal cancer	Lymph node metastasis in rectal cancer	Central depression	32/76 = 42%	3.00 (2.10–4.28)	Weak
Rectal cancer	Synchronous metastasis in rectal cancer	MRI-detected extramural vascular invasion (mrEMVI)	212/804 = 26%	5.65 (2.12–15.05)	Weak
Small rectal NETs	Lymph node metastasis in small rectal NETs treated by local excision	Lymphatic invasion	73/493 = 15%	5.54 (0.02–1752.46)	No association
Rectal cancer	Lymph node metastasis in rectal cancer	Vascular invasion	46/168 = 27%	5.86 (0.77–44.62)	No association
Small rectal NETs	Lymph node metastasis in small rectal NETs treated by local excision	Vascular invasion	75/211 = 36%	3.63 (0.05–268.57)	No association
pT1 CRC	Lymph node metastasis in pT1 CRC patients who underwent additional surgeries after an endoscopic resection	Poor or moderate differentiation	122/209 = 58%	3.77 (1.12–123.16)	No association
**Biomarker**					
CRC	Lymph node metastasis in CRC	Downregulated E-cadherin expression	829/1573 = 53%	0.49 (0.34–0.72)	Highly suggestive
CRC	Hepatic metastasis (distant) in CRC	Circulating tumor cells	103/310 = 33%	6.38 (2.67–15.26)	Suggestive
CRC	Lymph node metastasis in CRC	Low MUC2 expression level	613/1335 = 46%	1.42 (1.19–1.69)	Suggestive
CRC	Distant metastasis in CRC	Downregulated E-cadherin expression	509/1027 = 50%	0.45 (0.23–0.91)	Weak
CRC	Lymph node metastasis in CRC	Circulating tumor cells	797/1802 = 44%	1.62 (1.17–2.23)	Weak
CRC	Lymph node metastasis in CRC	p16 protein expression	482/800 = 60%	0.50 (0.30–0.84)	Weak
CRC	Distant metastasis in CRC	Cyclin D1 overexpression	952/1515 = 63%	0.60 (0.36–0.99)	Weak
CRC	Distant metastasis in CRC	β-catenin overexpression in the nucleus	283/531 = 53%	0.48 (0.29–0.79)	Weak
CRC	Lymph node metastasis in CRC	CD147 expression	603/815 = 74%	1.41 (0.39–5.01)	No association
CRC	Distant metastasis in CRC	CD147 expression	405/538 = 75%	2.32 (1.34E−06 to 4.03E+06)	No association
CRC	Lymph node metastasis in CRC	CD133 expression	550/1629 = 34%	1.15 (0.82–1.62)	No association
CRC	Distant metastasis in CRC	CD133 expression	300/1064 = 28%	1.54 (0.39–6.09)	No association
CRC	Lymph node metastasis in CRC	HER-2 immunohistochemical expression	440/1289 = 34%	1.90 (0.90–4.02)	No association
**Genetic risk factor**					
CRC	Lymph node metastasis in CRC	BRAF mutation	736/1142 = 64%	0.75 (0.49–1.14)	No association
CRC	Lymph node metastasis in CRC	RASSF1A promoter methylation	100/184 = 54%	1.61 (0.16–16.16)	No association
CRC	Distant metastasis in CRC	RASSF1A promoter methylation	153/417 = 37%	2.57 (0.64–10.24)	No association
**Demographic risk factor**					
pT1 CRC	Lymph node metastasis in pT1 CRC	Female gender	465/1329 = 35%	2.23 (0.78–6.42)	No association

*Abbreviation*: *CI* confidence interval, *CRC* colorectal cancer, *NET* neuroendocrine tumor

### Meta-analyses of risk factors for CRC recurrence

Overall, 20 eligible meta-analyses of observational studies investigating risk factors for CRC recurrence were identified (Additional file 1: Table S4). More than one meta-analysis was conducted for three risk factors (Additional file 1: Table S5). The direction of the summary effect size and the presence of nominal statistical significance (*p* < 0.05) of the reported associations between the same risk factor and the same outcome in overlapping meta-analyses were concordant for two (67%) risk factors (Additional file 1: Table S5).

A total of 17 unique meta-analyses with available data were retained for further analysis (Additional file 1: Table S7). The median number of included component studies was six (range 2–26), the median number of the total population was 2773 (range 252–39,745), and the median number of events was 551 (range 57–3675). The meta-analyses reported a wide range of risk factors (Additional file 1: Table S7): five histopathological risk factors (29%), two biomarkers (12%), one genetic risk factor (6%), five clinical risk factors (29%), one comorbidity (6%), and three anthropometric indices (18%). Overall, 11 (65%) of 17 unique meta-analyses reported effect sizes at *p* < 0.05 (Table 2).

**Table 2:** Summary of evidence credibility assessment of 17 unique meta-analyses of observational studies investigating the associations between risk factors and CRC recurrence

Population	Outcome	Risk factor	Risk factor prevalence	Effect size (95% CI)	Evidence classification
**Histopathological risk factor**					
CRC	Overall recurrence in CRC	Tumor budding	802/2773 = 29%	5.50 (3.65–8.29)	Highly suggestive
CRC	Overall recurrence in CRC	Extranodal extension (ENE)	376/877 = 43%	2.07 (1.65–2.61)	Highly suggestive
Rectal cancer	Local recurrence in rectal cancer	Perineural invasion (PNI)	336/1700 = 20%	3.21 (2.33–4.44)	Highly suggestive
Rectal cancer	Distant metastatic recurrence in rectal cancer	MRI-detected extramural vascular invasion (mrEMVI)	350/1262 = 28%	3.91 (2.61–5.86)	Highly suggestive
CRC	Local recurrence in CRC	Intramural vascular invasion (IMVI)	137/503 = 27%	1.55 (0.11–21.28)	No association
**Biomarker**					
CRC	Overall recurrence in CRC	Absence of peritoneal free tumor cells in pre-resection	524/593 = 88%	0.38 (0.16–0.91)	Weak
CRC	Overall recurrence in CRC	Absence of peritoneal free tumor cells in post-resection	214/252 = 85%	0.07 (0.02–0.21)	Weak
**Genetic risk factor**					
CRC	Overall recurrence in CRC	PTGS2 (also known as COX-2)	787/1516 = 52%	2.78 (1.76–4.40)	Suggestive
**Clinical risk factor**					
CRC	Local recurrence in CRC	Anastomotic leakage (AL)	3929/39,745 = 10%	1.90 (1.48–2.43)	Suggestive
Rectal cancer	Local recurrence in rectal cancer	Anastomotic leakage (AL)	1300/13,665 = 10%	1.61 (1.25–2.08)	Suggestive
CRC	Distant recurrence in CRC	Anastomotic leakage (AL)	865/10,392 = 8%	1.20 (0.94–1.52)	No association
Rectal cancer	Distant recurrence in rectal cancer	Anastomotic leakage (AL)	566/5221 = 11%	1.06 (0.72–1.58)	No association
Colon cancer	Local recurrence in colon cancer	Anastomotic leakage (AL)	91/1990 = 5%	2.19 (0.55–8.68)	No association
**Comorbidity**					
CRC	Overall recurrence in CRC	Diabetes	429/4979 = 9%	1.26 (0.70–2.30)	No association
**Anthropometric indices**					
CRC	Overall recurrence in CRC	Underweight	2752/17,636 = 16%	1.13 (1.05–1.21)	Weak
CRC	Overall recurrence in CRC	Obese	6362/21,246 = 30%	1.07 (1.02–1.13)	Weak
CRC	Overall recurrence in CRC	Overweight	13,225/28,109 = 47%	1.00 (0.96–1.05)	No association

*Abbreviation*: *CI* confidence interval, *CRC* colorectal cancer

No risk factor presented convincing evidence (Additional file 1: Table S10). In addition, four of 11 probed risk factors with *p* < 0.05 had an effect size suggesting ≥ 3-fold change in the odds of the outcome (Table 2).

### Methodological quality and risk of bias assessment

We assessed the methodological quality of 10 publications that included 16 meta-analyses of observational studies that statistically represented at least 3-fold changes in the odds of the outcome, using the AMSTAR 2.0 checklist (Additional file 1: Table S13). All assessed studies had more than one critical flaw [usually in items 2 (14/16, 88%), 7 (16/16, 100%), and 13 (13/16, 81%)] and several non-critical flaws [usually in items 3 (11/16, 69%), 10 (16/16, 100%), and 12 (16/16, 100%)]. It should be noted that all assessed meta-analyses had critically low quality. Therefore, the credibility of the available evidence should be interpreted with caution.

### Sensitivity analysis of redefying the disease outcome groups

We performed a sensitivity analysis to include individual component studies investigating risk factors for *metastasis at presentation* and re-evaluated the credibility of the evidence (Additional file 1: Table S11). A total of 16 unique meta-analyses including 67 (27%) component studies were retained and investigated. The remaining 185 (73%) studies did not illustrate when metastasis was present (i.e., at diagnosis or after a disease-free period) and therefore could not be included in this sensitivity analysis. Based on the pre-defined criteria, no risk factor presented convincing evidence.

Similarly, a sensitivity analysis was performed to include individual component studies investigating risk factors for *local or distant recurrence* (Additional file 1: Table S12). A total of 13 unique meta-analyses composed of 81 (58%) component studies (including five meta-analyses investigating distant metastasis after a period of being disease-free) were retained and investigated. The remaining 59 (42%) studies did not separate local or distant recurrence and therefore could not be included in our sensitivity analysis. Furthermore, no risk factor presented convincing evidence (Additional file 1: Table S12).

### Comparative cross-assessment between risk factors evaluated in the umbrella review and risk predictors applied in existing prediction models

#### Prediction models for CRC metastasis

Twelve prognostic models have been developed for prediction of CRC metastasis [22–33] (Table 3). The median number of included predictors was four (range 3–9), and 27 unique predictors were included in at least one model. Cancer stage (*N* = 9, 75%) was the most commonly used predictor variable in the 12 prognostic models. Other common predictors included histopathological risk factors such as positive lymph nodes (*N* = 3, 25%), tumor grade or differentiation (*N* = 2, 17%), and tumor histological type (*N* = 3, 25%); biomarker-carcinoembryonic antigen (CEA) (*N* = 3, 25%); age (*N* = 3, 25%); gender (*N* = 2, 17%); and clinical treatment such as surgery, chemotherapy, and radiotherapy (*N* = 3, 25%). Five models (42%) performed internal validation, and four models (33%) were validated in external datasets.

**Table 3:** Risk prediction models for CRC metastasis

Author, year	Country	Study design	Population	Outcome	Prediction time horizon	Sample size (development/validation)	Predictors	Model performance (95%CI)	Model presentation	Internal validation
*Macias-Garcia, 2015 [22]	Spain	D	Submucosal invasive (T1) CRC	Lymph node metastasis	NA	97	Sessile morphology	AUC 0.90 (0.81–0.99)	Risk score	NA
							Tumor differentiation			
							Infiltrative growth pattern			
							Lymphoid infiltrate			
Taylor, 1990 [23]	UK	D	CRC	Liver metastasis	10 years	134	Sex	Sensitivity 0.74, specificity 0.62	Formula	NA
							ALP			
							Dukes B			
							Dukes C			
Segelman, 2014 [24]	Sweden	D + IV	CRC (I–III)	Peritoneal metastasis	3 years	colon 5348/rectal 2696	Age	C statistic: colon 0.80, rectal 0.78; calibration plot	Calculator	Bootstrapping
							Cancer location			
							pT stage			
							pN stage			
							No. of lymph nodes examined			
							Preoperative radiotherapy radicality			
							Type of surgery			
							Adjuvant chemotherapy			
*Huang, 2016 [25]	China	D + IV	CRC	Lymph node metastasis	NA	266/60	Radiomics signature	C statistic 0.736 (0.759–0.766); Hosmer–Lemeshow test: *p* = 0.916	Nomogram	Random split
							CEA			
							CT-reported LN status			
*Hu, 2019 [26]	China	D + IV	CRC patients with indeterminate pulmonary nodules	Lung metastasis	NA	136/58	Chronicity (synchronous nodule or metachronous lesion)	AUC 0.929 (0.885–0.974); calibration plot	Nomogram	Random split
							Rad-score			
							pN stage			
*Xu, 2019 [27]	China	D + IV	CRC	Synchronous bone metastasis	NA	41,902/13,967	Cancer location	AUC 0.903; sensitivity 0.851; specificity 0.845	Risk score	Random split
							Tumor differentiation			
							Cancer histological type			
							CEA			
							pN stage			
							Brain metastasis			
							Liver metastasis			
							Lung metastasis			
*JR, 2019 [28]	Korea	D + EV	Submucosal invasive (T1) CRC	Lymph node metastasis	NA	833/722	Histologic grade	AUC 0.812 (0.770–0.855); Hosmer–Lemeshow test: *p* = 0.737	Nomogram	NA
							Submucosal invasion			
							Vascular invasion			
							Tumor budding			
Beumer, 2014 [29]	Netherlands	D + EV	CRC	Distant metastasis	5 years	50/43	MiR25/miR339	AUC 0.80 (0.67–0.93); calibration plot	Nomogram	NA
							AJCC stage			
							Age at surgery			
							Sex			
*Wang, 2017 [30]	China	D	Colon cancer	Peritoneal metastasis	NA	1417	Age	ROC 0.753	Nomogram	NA
							pT stage			
							Lesion not traversable			
							Infiltrative growth pattern			
							Tumor size			
							CEA			
							Histopathologic type of mucinous or signet ring cell adenocarcinoma			
Gijn, 2015 [31]	Netherlands	D + IV	Rectal cancer (tis–III)	Metastasis	5 years	2172	Distance from the anal verge	C statistic 0.761 (0.740–0.784); Hosmer–Lemeshow test: *p* = 0.82	Nomogram	Cross-validation
							pT stage			
							pA stage			
							pN stage			
							Surgery type			
							Residual tumor status			
Valentini, 2011 [32]	Belgium	D + EV	Rectal cancer (II–III)	Metastasis	5 years	3458	pT stage	External C statistic 0.73 (0.68–0.77); Wald statistic: *p* = 0.057	Nomogram	Random split
							pN stage			
							Surgery type			
							Adjuvant chemotherapy			
Sun, 2017 [33]	China	D + EV	Rectal cancer (I–III)	Distant metastasis	5 years	425/97	CRM	C statistic 0.70 (0.64–0.75)/0.71 (0.62–0.81); calibration plot	Nomogram	NA
							IMA nodes			
							AJCC stage			

Reproduced from He et al. [21]

*Abbreviations*: *D* model development, *D + IV* model development with internal validation, *D + EV* model development with external validation, *AJCC* American Joint Committee on Cancer, *ALP* alkaline phosphatase, *AUC* area under the curve, *CEA* carcinoembryonic antigen, *CRC* colorectal cancer, *CRM* circumferential resection margin, *IMA* inferior mesenteric artery, *NA* non-available, *miRNA* microRNA

*Six recently developed prediction models were additionally included, and data was extracted based on the previous criteria

We conducted a cross-assessment between these predictors and 34 risk factors that were evaluated in our umbrella review. Six of 27 unique predictors (*tumor budding*, *tumor differentiation*, *tumor size*, *vascular invasion*, *submucosal invasion*, and *sex*) were evaluated in the umbrella review (Table 5). The associated ORs for these six risk factors varied from 2.23 to 6.76, and four of them (67%) corresponded to ≥ 3-fold change in the odds of the outcome. Of the remaining 28 risk factors that were not employed in prediction models, ORs varied from 0.45 to 6.78, and 13 (46%) represented ≥ 3-fold change in the odds of the outcome.

In addition, we compared the overlapping outcomes to investigate whether prediction models had included influential risk factors (those presented convincing evidence or with 3-fold change in the odds of the outcome) when they predicted the same outcomes as those evaluated in the umbrella review (Table 6). In total, four overlapping outcomes were found in this cross-assessment (*LNM in pT1 CRC*, *LNM in CRC*, *hepatic metastasis in CRC*, and *distant metastasis in CRC*). For only one outcome (*LNM in pT1 CRC*), two prognostic models [22, 28] included four risk predictors that were also evaluated in the umbrella review, two of which corresponded to ≥ 3-fold change in the odds of the outcome (*tumor budding*, *tumor differentiation*).

#### Prediction models for CRC recurrence

Twelve prognostic models [31, 32, 34–43] were developed for prediction of CRC recurrence (Table 4). The median number of risk predictors applied in 12 prognostic models was five (range 2–8), and 25 unique predictors were included in at least one model. AJCC (TNM) cancer stage was the predictor variable (*N* = 7, 58%) used in the majority of CRC recurrence risk prediction models. Other common predictor variables included histopathological risk factors such as positive lymph nodes (*N* = 5, 42%), tumor grade or differentiation (*N* = 4, 33%), and tumor size (*N* = 4, 33%); biomarker-CEA (*N* = 4, 33%); cancer location (*N* = 4, 33%); and clinical treatment such as surgery, chemotherapy, radiotherapy, and transfusion (*N* = 5, 42%). Two models (17%) performed internal validation, and 4 models (33%) were validated in external datasets.

**Table 4:** Risk prediction models for CRC recurrence

Author, year	Country	Study design	Population	Outcome	Prediction time horizon	Sample size (development/validation)	Predictors	Model performance (95%CI)	Model presentation	Internal validation
Hoshino, 2016 [34]	Japan	D	CRC (II)	Overall recurrence	5 years	4167	Sex	C statistic 0.64; calibration plot	Nomogram	NA
							CEA			
							Tumor location			
							Tumor depth			
							Lymphatic invasion			
							Venous invasion			
							No. of positive lymph nodes			
Peng, 2010 [37]	China	D	CRC (II–III)	Overall recurrence	3 years	95	AJCC stage	AUC 0.75	Formula	NA
							Genetic score			
Ying, 2014 [38]	China	D	CRC (I–III, curative resection)	Overall recurrence	3 years	205	Tumor size	C statistic 0.810/0.890/0.802	Nomogram	NA
							Tumor differentiation			
							AJCC stage			
							NLR			
							Chemotherapy			
Zakaria, 2007 [39]	Japan	D	CRC (liver metastasis + hepatectomy)	Overall recurrence	5 years	662	Hepatoduodenal	C statistic 0.61 (0.57–0.64)/0.58 (0.550.61)	Nomogram	NA
							Lymph node status			
							Transfusions			
							Primary cancer			
							Regional lymph node			
							No. of metastasis			
Tian, 2017 [36]	China	D	CRC	Overall recurrence	3 years	556	Gene signature	AUC 0.921 (0.869–0.972); calibration plot	Nomogram	NA
							AJCC stage			
							Tumor differentiation			
*Kim, 2018 [40]	Korea	D + IV	CRC (I)	Overall recurrence	5 years	1538	Sex	C statistic 0.71; calibration plot	Nomogram	NA
							Tumor location			
							pT stage			
							LVI			
							Tumor size			
*Miyoshi, 2016 [41]	Japan	D + EV	CRC (IV with liver and/or lung metastases)	Overall recurrence	5 years	113	Preoperative CEA	C statistic 0.631	Nomogram	NA
							Tumor location			
							Tumor invasion			
							Lymph node metastasis			
							Synchronous metastatic lesions			
*Saso, 2018 [42]	Japan	D + EV	Colon cancer (II)	Overall recurrence	5 years	352/213	CEA level	C statistic 0.675; external C statistic 0.552	Nomogram	NA
							Tumor invasion			
							Lymphatic invasion			
							Venous invasion			
Renfro, 2014 [35]	USA	D + EV	Colon cancer (III)	Overall recurrence	5 years	15,995/1903	Sex	C statistic 0.65; calibration plot	Nomogram	NA
							BMI			
							PS			
							T stage			
							Lymph node ratio			
							Grade			
							Tumor location			
							Treatment			
Hida, 2017 [43]	Japan	D	Rectal cancer (II–III)	Overall recurrence	2 years	792	Tumor differentiation	AUC 0.831	Formula	NA
							Depth			
							Lymph node			
							Surgery			
							Postoperative complication			
							Tumor height			
							CEA			
Gijn, 2015 [31]	Netherlands	D + IV	Rectal cancer (tis-III)	Local recurrence	6 years	1823	Distance from the anal verge	C statistic 0.787 (0.761–0.814); Hosmer–Lemeshow test: *p* = 0.68	Nomogram	Cross-validation
							pT stage			
							pN stage			
							pM stage			
							Surgery type			
							Residual tumor status			
							Radiotherapy			
Valentini, 2011 [32]	Belgium	D + EV	Rectal cancer (II–III)	Local recurrence	5 years	3458	pT stage	External C statistic 0.68 (0.59–0.76); Wald statistic: *p* = 0.064	Nomogram	Random split
							cT stage			
							pN stage			
							Age			
							Concomitant chemotherapy			
							Adjuvant chemotherapy			

Reproduced from He et al. [21]

*Abbreviations*: *D* model development, *D + IV* model development with internal validation, *D + EV* model development with external validation, *AJCC* American Joint Committee on Cancer, *AUC* area under the curve, *BMI *body mass index,  *CEA* carcinoembryonic antigen, *CRC* colorectal cancer, *LVI* lymph vascular invasion, *NA* non-available, *NLR* neutrophil to lymphocyte ratio, *PS *performance status

*Three recently developed prediction models were additionally included, and data was extracted based on the previous criteria

In our cross-evaluation, five of 25 unique predictors (*intramural vascular invasion*, *extramural vascular invasion*, *being underweight*, *being overweight*, and *being obese*) were evaluated in the umbrella review (Table 5). The associated ORs for these five factors varied from 1.00 to 3.91, and only one (20%) (*extramural vascular invasion*) corresponded to ≥ 3-fold change in the odds of the outcome. Of the remaining 12 factors evaluated in the umbrella review, ORs varied from 0.07 to 5.50, and three (25%) represented ≥ 3-fold change in the odds of the outcome.

**Table 5:** Cross-assessment of the same risk factors and risk predictors

Risk factor/risk predictor	Outcome evaluated in the umbrella review	Risk factor prevalence	Effect size (95% CI)^**a**^	Credibility assessment	Outcome in the risk prediction models	Effect size (95% CI)^**b**^	Model performance
**CRC metastasis**							
**Histopathological risk factor**							
Vascular invasion	Lymph node metastasis in pT1 CRC	330/1731 = 19%	2.73 (1.98–3.78)	Convincing	Lymph node metastasis in pT1 CRC	8.45 (4.56–15.66)	AUC 0.812 (0.770–0.855); Hosmer–Lemeshow test: *p* = 0.737 (55)
	Lymph node metastasis in rectal cancer	46/168 = 27%	5.86 (0.77–44.62)	No association			
	Lymph node metastasis in small rectal NETs treated by local excision	75/211 = 36%	3.63 (0.05–268.57)	No association			
Tumor budding	Lymph node metastasis in pT1 CRC	2401/10,128 = 24%	6.39 (5.23–7.80)	Highly suggestive	Lymph node metastasis in pT1 CRC	1.70 (1.03–2.80)	AUC 0.812 (0.770–0.855); Hosmer–Lemeshow test: *p* = 0.737 (55)
	Lymph node metastasis in CRC	1955/6739 = 29%	4.96 (3.97–6.19)	Highly suggestive			
Tumor differentiation	Lymph node metastasis in pT1 CRC	94/2722 = 4%	5.61 (2.90–10.83)	Suggestive	Lymph node metastasis in pT1 CRC	11.77 (0.77–179.83)	AUC 0.90 (0.81–0.99) (49)
	Lymph node metastasis in pT1 CRC patients who underwent additional surgeries after an endoscopic resection	122/209 = 58%	3.77 (1.12–123.16)	No association	Synchronous bone metastasis	1.69 (1.22–2.32)	AUC 0.903; sensitivity 0.851; specificity 0.845 (54)
Submucosal invasion ≥ 1 mm	Lymph node metastasis in pT1 CRC	2389/2922 = 82%	2.95 (1.39–6.27)	Weak	Lymph node metastasis in pT1 CRC	2.14 (1.19–3.86)	AUC 0.812 (0.770–0.855); Hosmer–Lemeshow test: *p* = 0.737 (55)
Tumor size > 1 cm	Lymph node metastasis in rectal cancer	203/348 = 58%	6.76 (3.25–14.04)	Highly suggestive	Peritoneal metastasis in colon cancer	1.04 (1.00–1.09)	ROC 0.753 (57)
**Demographic risk factor**							
Sex/gender	Lymph node metastasis in pT1 CRC	465/1329 = 35%	2.23 (0.78–6.42)	No association	Liver metastasis in CRC	NA	Sensitivity 0.74; specificity 0.62 (50)
					Distant metastasis in CRC	1.40 (0.46–4.28)	AUC 0.80 (0.67–0.93); calibration plot (56)
**CRC recurrence**							
**Histopathological risk factor**							
Vascular invasion (intramural)	Local recurrence in CRC	137/503 = 27%	1.55 (0.11–21.28)	No association	Overall recurrence in stage II CRC	1.30 (1.07–1.58)	C statistic 0.64; calibration plot (61)
Vascular invasion (extramural)	Distant metastatic recurrence in rectal cancer	350/1262 = 28%	3.91 (2.61–5.86)	Highly suggestive	Overall recurrence in stage II colon cancer	2.48 (1.22–5.57)	C statistic 0.675; external C statistic 0.552 (68)
**Anthropometric indices**							
BMI (underweight)	Overall recurrence in CRC	2752/17,636 = 16%	1.13 (1.05–1.21)	Weak	Overall recurrence in stage III colon cancer	NA	C statistic 0.65; calibration plot (69)
BMI (overweight)	Overall recurrence in CRC	13,225/28,109 = 47%	1.00 (0.96–1.05)	No association			
BMI (obese)	Overall recurrence in CRC	6362/21,246 = 30%	1.07 (1.02–1.13)	Weak			

*Abbreviations*: *AUC* area under the curve, *BMI* body mass index, *CI* confidence interval, *CRC* colorectal cancer, *NA* non-available

^a^Effect size (95% CI), effect size from the umbrella review

^b^Effect size (95% CI), effect size from the risk prediction models

In relation to overlapping outcomes, only one outcome (*overall recurrence in CRC*) was identified (Table 6). However, the prognostic model [36] included risk predictors that were not evaluated in the umbrella review (*cancer stage*, *tumor differentiation*, and *gene signature*). Meanwhile, within the evaluated nine risk factors for overall recurrence in CRC that were not employed as predictors in this model, only two influential risk factors (*tumor budding*, *absence of peritoneal free tumor cells in post-resection*) had ≥ 3-fold change in the odds of the outcome.

**Table 6:** Cross-assessment of the same outcomes with their corresponding risk factors and predictors

Overlapping outcomes	Risk factor	Risk factor prevalence	Effect size (95% CI)^**a**^	Credibility assessment	Risk predictor	Effect size (95% CI)^**b**^	Model performance
**CRC metastasis**							
Lymph node metastasis in pT1 CRC	Vascular invasion	330/1731 = 19%	2.73 (1.98–3.78)	Convincing	Vascular invasion	8.45 (4.56–15.66)	AUC 0.812 (0.770–0.855); Hosmer–Lemeshow test: *p* = 0.737 (55)
	Submucosal invasion ≥ 1 mm	2389/2922 = 82%	2.95 (1.39–6.27)	Weak	Submucosal invasion ≥ 1 mm	2.14 (1.19–3.86)	
	Tumor budding	2401/10,128 = 24%	6.39 (5.23–7.80)	Highly suggestive	Tumor budding	1.70 (1.03–2.80)	
					Histologic grade	7.89 (2.89–21.52)	
	Tumor differentiation	94/2722 = 4%	5.61 (2.90–10.83)	Suggestive	Tumor differentiation	11.77 (0.77–179.83)	AUC 0.90 (0.81–0.99) (49)
	Lymphatic invasion	906/3347 = 27%	6.78 (5.29–8.69)	Highly suggestive	Infiltrative growth pattern	31.91 (2.37–428.36)	
	Lymphovascular invasion	340/1695 = 20%	4.81 (3.14–7.36)	Suggestive	Lymphoid infiltrate	28.75 (2.13–388.37)	
	Gender	465/1329 = 35%	2.23 (0.78–6.42)	No association	Sessile morphology	4.88 (0.81–29.3)	
Lymph node metastasis in CRC	Tumor budding	1955/6739 = 29%	4.96 (3.97–6.19)	Highly suggestive	CT-reported lymph node status	1.69 (1.05–2.75)	C statistic 0.736 (0.759–0.766); Hosmer–Lemeshow test: *p* = 0.916 (52)
	Downregulated E-cadherin expression	829/1573 = 53%	0.49 (0.34–0.72)	Highly suggestive	Radiomics signature	5.48 (3.03–9.91)	
	Low MUC2 expression level	613/1335 = 46%	1.42 (1.19–1.69)	Suggestive	CEA	1.71 (1.04–2.83)	
	Circulating tumor cells	797/1802 = 44%	1.62 (1.17–2.23)	Weak			
	p16 protein expression	482/800 = 60%	0.50 (0.30–0.84)	Weak			
	CD147 expression	603/815 = 74%	1.41 (0.39–5.01)	No association			
	CD133 expression	550/1629 = 34%	1.15 (0.82–1.62)	No association			
	HER-2 immunohistochemical expression	440/1289 = 34%	1.90 (0.90–4.02)	No association			
	BRAF mutation	736/1142 = 64%	0.75 (0.49–1.14)	No association			
	RASSF1A promoter methylation	100/184 = 54%	1.61 (0.16–16.16)	No association			
Hepatic metastasis in CRC	Circulating tumor cells	103/310 = 33%	6.38 (2.67–15.26)	Suggestive	Duke B/C	NA	Sensitivity 0.74, specificity 0.62 (50)
					ALP	NA	
					Sex/gender	NA	
Distant metastasis in CRC	Downregulated E-cadherin expression	509/1027 = 50%	0.45 (0.23–0.91)	Weak	AJCC stage	1.27 (0.25–6.38)	AUC 0.80 (0.67–0.93); calibration plot (56)
	Cyclin D1 overexpression	952/1515 = 63%	0.60 (0.36–0.99)	Weak	MiR25/MiR339	2.92 (0.98–8.64)	
	β-catenin overexpression in the nucleus	283/531 = 53%	0.48 (0.29–0.79)	Weak	Age at surgery	1.10 (0.20–6.03)	
	CD147 expression	405/538 = 75%	2.32 (1.34E−06 to 4.03E+06)	No association	Sex/gender	1.40 (0.46–4.28)	
	CD133 expression	300/1064 = 28%	1.54 (0.39–6.09)	No association			
	RASSF1A promoter methylation	153/417 = 37%	2.57 (0.64–10.24)	No association			
**CRC recurrence**							
Overall recurrence in CRC	Tumor budding	802/2773 = 29%	5.50 (3.65–8.29)	Highly suggestive	AJCC stage	NA	AUC 0.921 (0.869–0.972); calibration plot (65)
	Extranodal extension (ENE)	376/877 = 43%	2.07 (1.65–2.61)	Highly suggestive	Tumor differentiation	NA	
	PTGS2 (COX-2)	787/1516 = 52%	2.78 (1.76–4.40)	Suggestive	Gene signature	NA	
	Absence of peritoneal free tumor cells in pre-resection	524/593 = 88%	0.38 (0.16–0.91)	Weak			
	Absence of peritoneal free tumor cells in post-resection	214/252 = 85%	0.07 (0.02–0.21)	Weak			
	Underweight	2752/17,636 = 16%	1.13 (1.05–1.21)	Weak			
	Obese	6362/21,246 = 30%	1.07 (1.02–1.13)	Weak			
	Overweight	13,225/28,109 = 47%	1.00 (0.96–1.05)	No association			
	Diabetes	429/4979 = 9%	1.26 (0.70–2.30)	No association			

*Abbreviations*: *ALP* alkaline phosphatase, *AUC* area under the curve, *BMI* body mass index, *CEA* carcinoembryonic antigen, *CI* confidence interval, *CRC* colorectal cancer, *NA* non-available, *miRNA* microRNA

^a^Effect size (95% CI), effect size from the umbrella review

^b^Effect size (95% CI), effect size from the risk prediction models

## Discussion

We initially synthesized and evaluated the evidence of risk factors for CRC metastasis and recurrence. Our study comprised 51 unique meta-analyses of observational studies investigating 34 risk factors for CRC metastasis and 17 risk factors for recurrence. We also conducted a sensitivity analysis of 29 unique meta-analyses of risk factors for CRC metastasis at presentation (*n* = 16), CRC local recurrence (*n* = 5), and CRC distant recurrence (*n* = 8) using a standardized categorization of the component studies. Furthermore, we updated synthesis of risk prediction models for CRC metastasis (*n* = 12) and recurrence (*n* = 12) and then conducted a cross-assessment of individual risk factors evaluated in the umbrella review and risk predictors included in existing prediction models, which allowed us to examine to what extent predictive models include the most influential factors.

### Main findings and interpretation of the umbrella review

#### Meta-analyses for CRC metastasis

According to our pre-defined criteria for assessing the credibility of the evidence, only one risk factor was classified as convincing (*vascular invasion for LNM in pT1 CRC*), reflecting strong statistical significance and no hints of bias. Many studies have demonstrated that the invasion of blood vessels leading to tumor cell dissemination and metastasis is a strong risk factor for disease prognosis, which is in line with our umbrella review [44, 45]. Based on our findings, a large proportion of studies (17/25, 68%) investigated lymphatic and vascular invasion as separate risk factors, while 32% of studies categorized them jointly as lymphovascular invasion. It has been shown though that the predictive ability of lymphovascular invasion is lower than that of vascular invasion [46].

Twelve (35%) of 34 probed risk factors for metastasis had an effect size suggesting ≥ 3-fold change in the odds of the outcome with *p* < 0.05. Four of these risk factors (*lymphatic invasion for LNM in pT1 CRC*; *tumor budding for LNM in pT1 CRC*; *tumor budding for LNM in all stage CRC*; *tumor size > 1 cm for LNM in rectal cancer*) were classified as highly suggestive. As discussed above, *lymphatic invasion* could be an indicator of tumor cells metastasizing to lymph nodes. This finding agrees with three recently published studies manifesting that lymphatic invasion is causally associated with the risk of LNM in CRC [47–49]. *Tumor budding* is recognized as a negative prognostic risk factor for LNM in CRC, and our findings are concordant with previous studies [50–52]. Individual component studies vary in their definitions of tumor budding (e.g., how many cancer cells comprise a tumor bud, and how many buds signify tumor budding) and vary in the pathologic staining methods to detect tumor budding (e.g., hematoxylin and eosin [H&E], immunohistochemistry [IHC]). Furthermore, a systematic review summarized pathologic methods to detect tumor budding and revealed that all studies even when utilizing different methods showed that tumor budding increases the risk of CRC metastasis [53]. Notably, substantial between-study heterogeneity (*I*^2^ > 50%) was found in the meta-analysis investigating tumor budding for LNM in all CRC stages, indicating that this association needs to be interpreted with caution. The observed heterogeneity may be influenced by the inclusion of different tumor stages. Finally, *tumor size > 1 cm* is associated with an increased risk of LNM in rectal cancer. This largely agrees with the European Society for Medical Oncology (ESMO) clinical practice guideline manifesting that a rectal lesion less than 1 cm has a lower risk of metastasis, and therefore, local excision (TEM) is suggested [54].

#### Meta-analyses for CRC recurrence

In regard to 17 probed risk factors for CRC recurrence, four (24%) had an effect size suggesting ≥ 3-fold change in the odds of the outcome with *p* < 0.05. None of them presented convincing evidence. Three (*tumor budding for overall recurrence in CRC*; *perineural invasion [PNI] for local recurrence in rectal cancer*; *MRI-detected extramural vascular invasion [mrEMVI] for distant metastatic recurrence in rectal cancer*) were classified as highly suggestive. Our findings suggest that *tumor budding* is a common highly suggestive risk factor for both CRC LNM and overall recurrence. However, there is a need for standardization of the histopathological definition of tumor budding [46]. Another histopathological risk factor, *PNI*, which is a common pathological feature in rectal cancer, strongly signifies local recurrence. Compared to colon cancer, PNI occurs more frequently in rectal cancer, since there is a cluster of intensive neural plexuses surrounding the pelvis in the rectum [55]. The National Comprehensive Cancer Network (NCCN) guidelines also suggest that patients with PNI positive are at higher risk of local recurrence [56]. However, there is no consensus in the definition of PNI positive, with two of the most frequently used definitions being SS-PNI (when tumor cells surround at least 33% of the nerve) and TS-PNI (when tumor cells surround any of the three layers of the nerve) [57–60]. Finally, we found that *mrEMVI* increases the risk of distant metastatic recurrence. EMVI is the venous invasion beyond the muscularis propria, which has long been recognized as a risk factor for distant recurrence [61–63]. The 5-point MRI-detected EMVI scoring system is precise for detecting this invasion, and it is recommended as a post-operation follow-up strategy in clinical settings [64]. In addition, a recently published meta-analysis is also in line with our findings, reporting that around 90% of patients with liver metastases are mrEMVI positive [65].

#### Sensitivity analysis

In our effort for a consistent definition of metastasis and recurrence, we re-categorized all the component studies to three distinct disease outcomes: metastasis at presentation, local recurrence, and distant recurrence. This could generate insight into metastasis and recurrence patterns and provide investigators and clinicians with a more comprehensive summary of risk factors for these CRC prognostic outcomes with clinical significance [66]. Our sensitivity analyses reported a dearth of convincing evidence. However, a total of 244 (62%) individual component studies were excluded from our sensitivity analyses due to missing information in relation to outcome definition.

### Cross-assessment between risk factors evaluated in the umbrella review and risk predictors applied in existing prediction models

We identified 24 CRC prognostic models for metastasis (*n* = 12) and recurrence (*n* = 12). The majority of risk prediction models applied an average of four to five predictor variables. The most commonly used predictors were clinic-histopathological (cancer stage, lymph node status) and demographic (gender, age) parameters. Seven models were validated internally and eight in external datasets, but none of the identified models conducted any impact studies. As for model presentation, the majority of models were nomograms (graphical prediction models), and the remaining models were presented as formulae, risk scores, and calculators.

In our cross-assessment, we investigated whether the identified prediction models had employed influential risk factors (those presented convincing evidence or with 3-fold change in the odds of the outcome) when they predicted the same outcomes as those that were evaluated in the umbrella review. Across 12 CRC metastasis risk prediction models, five models [22, 23, 25, 28, 29] were on the same outcomes (*LNM in pT1 CRC*, *LNM in CRC*, *hepatic metastasis in CRC*, and *distant metastasis in CRC*), with only two [22, 28] of these models (*on LNM in pT1 CRC*) including predictors also evaluated in the umbrella review. However, the models’ calibration was poorly reported, which made it difficult to assess the models’ predictive accuracy. Furthermore, one model [28] was externally validated to ensure the model’s applicability and generalizability, while the remaining one [22] did not undergo adequate validation to address its potential overfitting. In addition, the remaining three models [23, 25, 29] predicting LNM and DM in CRC applied other risk predictors such as cancer stage, CEA, and alkaline phosphatase (ALP) that were not evaluated in the umbrella review. We suggest that risk factors with strong associations with CRC prognosis, such as circulating tumor cells and microsatellite instability, should be employed following evidence-based methods.

Across the 12 CRC recurrence risk prediction models, only one model [36] was on an outcome that was also evaluated in the umbrella review (*overall recurrence in CRC*). Unfortunately, we did not find overlapping risk factors/predictors. We recommend tumor budding and absence of peritoneal free tumor cells in post-resection (≥ 3-fold change in the odds of the outcome) to be considered as predictors.

### Clinical implications and future research

Identifying and evaluating risk factors with substantial predictive value is of great clinical importance. Major clinical decisions are made taking into account expectations and formal or informal predictions about major outcomes. Accurate and valid risk prediction could assist with clinical decision-making in relation to the extent and mode of surgery and therapy. Ideally, adjuvant treatment would be targeted with precision to those most likely to benefit; those most at risk of CRC metastasis/recurrence may also have a higher absolute probability of benefit. The majority of patients do not benefit from additional therapy aimed at preventing locoregional or distant relapse before or after surgical resection, and yet they may be exposed to the attendant morbidity, cost, and false expectation of such therapy. Therefore, accurate and valid risk prediction which could impact clinical decision-making is crucial. In summary, this umbrella review provides an evidence classification that could help clinicians to judge the relative priority of risk factors/predictors’ impact on CRC prognosis and make clinical decisions based on more accurate and valid risk prediction.

Our findings suggest that efforts to address the limitations of the available evidence could be beneficial. Large-scale prospective studies are needed to generate evidence less prone to bias and allowing better predictive model building and validation. Standardizing the outcome definitions of CRC metastasis and recurrence could improve reporting of outcomes that have direct clinical relevance. Future risk prediction model research is encouraged to apply rigorous model construction processes and to integrate the most influential risk factors based on evidence-based methods.

### Strengths and limitations

The main strength of this study is that it provides a rigorous critical assessment of the published epidemiological evidence on risk factors of CRC metastasis and recurrence, based on pre-defined criteria in a transparent and systematic way [17, 18]. In addition, we updated the synthesis of CRC prognostic prediction models, and to our best knowledge, this is the first cross-assessment between individual risk factors and risk predictors applied in existing prediction models, to investigate whether influential risk factors are employed as predictors. Our findings provide a comprehensive evaluation of available evidence that can inform future research on risk factors for CRC prognostic outcomes and risk prediction models.

However, the following potential limitations should be considered. First, umbrella review comprises a synthesis of evidence from existing systematic reviews and meta-analyses [67]. Therefore, risk factors and risk predictors that were not systematically reviewed in the pre-existing literature are not included in this umbrella review. These may include some factors that are commonly used in predictive models, and it highlights the need to perform systematic reviews of the evidence for factors that might be routinely or frequently measured. Second, meta-analyses have common defects such as limited coverage of the literature search and low quality of the included studies [68, 69]. Third, this study only collected and evaluated evidence from systematic reviews and meta-analyses of observational studies published in peer-reviewed journals. This could limit the breadth of our results if research in gray literature, conference abstracts, and comments investigated risk factors that were not included in this umbrella review. Furthermore, 77% of meta-analyses included only retrospective studies.

Moreover, this study did not evaluate the quality of all individual component studies included in each meta-analysis because it is beyond the scope of an umbrella review. Instead, we performed a credibility evaluation and risk of bias assessment for meta-analyses that represented at least 3-fold changes in the odds of the outcome. Criteria for assessing the evidence from meta-analyses of observational studies applied in our umbrella review were based on pre-defined metrics whose limitations have been summarized [70–72]. For the outcomes that we studied, one is probably interested usually on whether the considered risk factors confer substantial predictive value, rather than whether they are causally related to the outcomes. We pre-specified a threshold for the magnitude of what might be a relatively large effect size (3-fold change in odds), but this is not absolute. The predictive value may depend also on how frequently a given factor is in the evaluated population. However, with one exception, all the factors evaluated concurrently in both risk factor meta-analyses and in predictive models were pretty common, with prevalence ranging from 16 to 82%.

We should also acknowledge that although we performed a sensitivity analysis to classify CRC metastasis at presentation, local or distant recurrence, a large proportion (62%) of individual component studies did not present enough information, such as the timing of metastasis in relation to initial diagnosis (i.e., synchronous or metachronous) and local or distant recurrence separately from overall recurrence. Finally, we did not evaluate risk factors relevant to clinical interventions such as surgery type, chemotherapy, radiotherapy, and transfusion. We also could not perform a complete comparison between risk factors evaluated in the umbrella review and risk predictors applied in existing prediction models because only 11 overlapping risk factors/predictors were identified.

## Conclusions

In this umbrella review, we synthesized and evaluated risk factors and risk prediction models of CRC metastasis and recurrence. A total of 51 unique risk factors were investigated, convincing evidence exists only for the association between vascular invasion and LNM, and even that is restricted to pT1 tumors. Furthermore, we also conducted a cross-assessment to evaluate individual risk factors and risk prediction models. Our findings emphasize the need for a more rigorous and systematic model construction process to integrate influential risk factors following evidence-based methods.

## Supplementary information

**Additional file 1: Table S1.** Search strategy. **Table S2.** A list of publications included in the umbrella review. **Table S3.** Quantitative synthesis of all 61 eligible meta-analyses of observational studies investigating the associations between risk factors and colorectal cancer metastasis. **Table S4.** Quantitative synthesis of all 20 eligible meta-analyses of observational studies investigating the associations between risk factors and colorectal cancer recurrence. **Table S5.** Overlapping meta-analyses of observational studies investigating the associations between the same risk factor and the same outcome. **Table S6.** Quantitative synthesis of 34 unique meta-analyses of observational studies investigating the associations between risk factors and colorectal cancer metastasis. **Table S7.** Quantitative synthesis of 17 unique meta-analyses of observational studies investigating the associations between risk factors and colorectal cancer recurrence. **Table S8.** Criteria for assessing the credibility of the evidence from meta-analyses of observational studies. **Table S9.** Summary of evidence credibility assessment of 34 unique meta-analyses of observational studies investigating the associations between risk factors and colorectal cancer metastasis. **Table S10.** Summary of evidence credibility assessment of 17 unique meta-analyses of observational studies investigating the associations between risk factors and colorectal cancer recurrence. **Table S11.** Sensitivity analysis of 16 unique meta-analyses of observational studies investigating the associations between risk factors and colorectal cancer metastasis (at presentation) and evidence credibility assessment. **Table S12.** Sensitivity analysis of 13 unique meta-analyses of observational studies investigating the associations between risk factors and colorectal cancer recurrence (local/ distant) and evidence credibility assessment. **Table S13.** Quality and risk of bias assessment (AMSTAR 2.0) for the evidence represented at least 3-fold changes in the odds of the outcome.

## Data Availability

Not applicable.

## Abbreviations

AMSTAR 2.0: Assessment of Multiple Systematic Reviews 2.0
ALP: Alkaline phosphatase
CEA: Carcinoembryonic antigen
CI: Confidence interval
CRC: Colorectal cancer
DL: DerSimonian and Laird
EMR: Endoscopic resection
ESMO: European Society for Medical Oncology
H&E: Hematoxylin and eosin
HKSJ: Hartung-Knapp-Sidik-Jonkman
HR: Hazard ratio
IHC: Immunohistochemistry
LNM: Lymph node metastasis
mrEMVI: MRI-detected extramural vascular invasion
NCCN: National Comprehensive Cancer Network
OR: Odds ratio
PNI: Perineural invasion
PRISMA-P: Preferred Reporting Items for Systematic Reviews and Meta-Analyses Protocols
RR: Risk ratio
TEM: Trans-anal microscopic surgery
