# Supplementary material for: Risk factors and risk prediction models for colorectal cancer metastasis and recurrence: an umbrella review of systematic reviews and meta-analyses of observational studies
Source: BMC Med. 2020 Jun 26;18:172. doi: 10.1186/s12916-020-01618-6 (PMC7318747; doi:10.1186/s12916-020-01618-6)
Supplement: Supplementary file 1 — Additional file 1: Table S1. Search strategy. Table S2. A list of publications included in the umbrella review. Table S3. Quantitative synthesis of all 61 eligible meta-analyses of observational studies investigating the associations between risk factors and colorectal cancer metastasis. Table S4. Quantitative synthesis of all 20 eligible meta-analyses of observational studies investigating the associations between risk factors and colorectal cancer recurrence. Table S5. Overlapping meta-analyses of observational studies investigating the associations between the same risk factor and the same outcome. Table S6. Quantitative synthesis of 34 unique meta-analyses of observational studies investigating the associations between risk factors and colorectal cancer metastasis. Table S7. Quantitative synthesis of 17 unique meta-analyses of observational studies investigating the associations between risk factors and colorectal cancer recurrence. Table S8. Criteria for assessing the credibility of the evidence from meta-analyses of observational studies. Table S9. Summary of evidence credibility assessment of 34 unique meta-analyses of observational studies investigating the associations between risk factors and colorectal cancer metastasis. Table S10. Summary of evidence credibility assessment of 17 unique meta-analyses of observational studies investigating the associations between risk factors and colorectal cancer recurrence. Table S11. Sensitivity analysis of 16 unique meta-analyses of observational studies investigating the associations between risk factors and colorectal cancer metastasis (at presentation) and evidence credibility assessment. Table S12. Sensitivity analysis of 13 unique meta-analyses of observational studies investigating the associations between risk factors and colorectal cancer recurrence (local/ distant) and evidence credibility assessment. Table S13. Quality and risk of bias assessment (AMSTAR 2.0) for the evidence represented at least 3-fold chan [file 12916_2020_1618_MOESM1_ESM.docx]

**SUPPLEMENTARY MATERIAL**

**Table S1**: Search strategy

**Table S2**: A list of publications included in the umbrella review

**Table S3**: Quantitative synthesis of all 61 eligible meta-analyses of observational studies investigating the associations between risk factors and colorectal cancer metastasis

**Table S4**: Quantitative synthesis of all 20 eligible meta-analyses of observational studies investigating the associations between risk factors and colorectal cancer recurrence

**Table S5**: Overlapping meta-analyses of observational studies investigating the associations between the same risk factor and the same outcome

**Table S6**: Quantitative synthesis of 34 unique meta-analyses of observational studies investigating the associations between risk factors and colorectal cancer metastasis

**Table S7**: Quantitative synthesis of 17 unique meta-analyses of observational studies investigating the associations between risk factors and colorectal cancer recurrence

**Table S8**: Criteria for assessing the credibility of the evidence from meta-analyses of observational studies

**Table S9**: Summary of evidence credibility assessment of 34 unique meta-analyses of observational studies investigating the associations between risk factors and colorectal cancer metastasis

**Table S10**: Summary of evidence credibility assessment of 17 unique meta-analyses of observational studies investigating the associations between risk factors and colorectal cancer recurrence

**Table S11**: Sensitivity analysis of 16 unique meta-analyses of observational studies investigating the associations between risk factors and colorectal cancer metastasis (at presentation) and evidence credibility assessment

**Table S12**: Sensitivity analysis of 13 unique meta-analyses of observational studies investigating the associations between risk factors and colorectal cancer recurrence (local/ distant) and evidence credibility assessment

**Table S13**: Quality and risk of bias assessment (AMSTAR 2.0) for the evidence represented at least 3-fold changes in the odds of the outcome

**Table S1: Search strategy**

| **PubMed:**  (((((("Risk Factors"[Mesh]) OR risk factor)) AND (("Colorectal Neoplasms"[Mesh]) OR ((((((((((((neoplas*) OR tumor*) OR tumour*) OR cancer*) OR carcinoma*) OR malignan*) OR adenocarcinoma*) OR adeno?carcinoma*) OR adenom*) OR CRC)) AND ((((((((((colorectal) OR colon) OR colonic) OR rectum) OR rectal) OR bowel*) OR intestine*) OR sigmoid) OR anus) OR anal)))) AND (("Prognosis"[Mesh]) OR prognos*)) OR (("Recurrence"[Mesh]) OR (((recurrences) OR recrudescence*) OR relapse*))) OR ((metastasis) OR metastas*))) AND ( ( Meta-Analysis[ptyp] OR systematic[sb] ) )) |
| --- |
| **Cochrane Library:**  1: MeSH descriptor: [Risk Factors] explode all trees  2: risk factor  3: 1 OR 2  4: (colorectal) OR (colon) OR (colonic) OR (rectum) OR (rectal) OR (bowel*) OR (intestine*) OR (sigmoid) OR (anus) OR (anal)  5: (neoplas*) OR (tumor*) OR (tumour*) OR (cancer*) OR (carcinoma*) OR (malignan*) OR (adenocarcinoma*) OR (adeno?carcinoma*) OR (adenom*) OR (CRC)  6: 4 AND 5  7: MeSH descriptor: [Colorectal Neoplasms] explode all trees  8: 6 OR 7  9: (metastasis) OR (metastas*)  10: MeSH descriptor: [Recurrence] explode all trees  11: (recurrences) OR (recrudescence*) OR (relapse*)  12: 10 OR 11  13: MeSH descriptor: [Prognosis] explode all trees  14: prognos*  15: 13 OR 14  16: 9 OR 12 OR 15  17: 3 AND 8 AND 16  18: (meta-analys*) OR (systematic review*)  19: 17 AND 18 |
| **Web of Science:**  1: TS= (risk factor*)  2: TS= (colorectal OR colon OR colonic OR rectum OR rectal OR bowel* OR intestine* OR sigmoid OR anus OR anal)  3: TS= (neoplas* OR tumor* OR tumour* OR cancer* OR carcinoma* OR malignan* OR adenocarcinoma* OR adeno?carcinoma* OR adenom* OR CRC)  4: TS= (metastasis OR metastas*)  5: TS= (recurrence* OR recrudescence* OR relapse*)  6: TS= (prognosis OR prognos*)  7: 4 OR 5 OR 6  8: TS= (meta-analys* OR systematic review*)  9: 1 AND 2 AND 3 AND 7 AND 8 |
| **EMBASE:**  1: risk factor/ or risk factors.mp.  2: ((colorectal or colon or colonic or rectum or rectal or bowel* or intestine* or sigmoid or anus or anal) adj2 (neoplas* or tumor* or tumour* or cancer* or carcinoma* or malignan* or adenocarcinoma* or adeno?carcinoma* or adenom* or CRC)).mp.  3: colorectal cancer/ or colon cancer/ or rectum cancer/ or sigmoid cancer/ or anus cancer/ or colorectal tumor/ or colon tumor/ or rectum tumor/ or anus tumor/ or colorectal carcinoma/ or colon carcinoma/ or rectum carcinoma/ or sigmoid carcinoma/ or anus carcinoma/  4: 2 OR 3  5: metastasis/ or metastas*.mp.  6: cancer recurrence/ or recurrence*.mp. or recrudescence*.mp. or relapse*.mp.  7: cancer prognosis/ or prognos*.mp.  8: 5 or 6 or 7  9: systematic review.mp. or “systematic review”/ or meta-analysis/ or meta- analysis.mp.  10: 1 and 4 and 8 and 9 |

**Table S2: A list of publications included in the umbrella review**

| **Systematic reviews** | |
| --- | --- |
| **CRC metastasis and risk factors** | |
| 1 | Christensen, T.D., Jensen, S.G., Larsen, F.O. and Nielsen, D.L., 2018. Systematic review: Incidence, risk factors, survival and treatment of bone metastases from colorectal cancer. *Journal of bone oncology.* |
| 2 | Christensen, T.D., Spindler, K.L.G., Palshof, J.A. and Nielsen, D.L., 2016. Systematic review: brain metastases from colorectal cancer—incidence and patient characteristics. *BMC cancer, 16*(1), p.260. |
| 3 | McDermott, F.D., Heeney, A., Courtney, D., Mohan, H. and Winter, D., 2014. Rectal carcinoids: a systematic review. *Surgical endoscopy, 28*(7), pp.2020-2026. |
| **CRC recurrence and risk factors** | |
| 4 | Honoré, C., Gelli, M., Francoual, J., Benhaim, L., Elias, D. and Goéré, D., 2017. Ninety percent of the adverse outcomes occur in 10% of patients: can we identify the populations at high risk of developing peritoneal metastases after curative surgery for colorectal cancer?. *International Journal of Hyperthermia*, *33*(5), pp.505-510. |
| 5 | Peng, J.Y., Li, Z.N. and Wang, Y., 2013. Risk factors for local recurrence following neoadjuvant chemoradiotherapy for rectal cancers. *World Journal of Gastroenterology: WJG, 19*(32), p.5227. |
| **Meta-analysis of observational studies** | |
| **CRC metastasis and risk factors** | |
| 6 | Beaton, C., Twine, C.P., Williams, G.L. and Radcliffe, A.G., 2013. Systematic review and meta‐analysis of histopathological factors influencing the risk of lymph node metastasis in early colorectal cancer. *Colorectal Disease, 15*(7), pp.788-797. |
| 7 | Bosch, S.L., Teerenstra, S., de Wilt, J.H., Cunningham, C. and Nagtegaal, I.D., 2013. Predicting lymph node metastasis in pT1 colorectal cancer: a systematic review of risk factors providing rationale for therapy decisions. *Endoscopy, 45*(10), pp.827-841. |
| 8 | Cappellesso, R., Luchini, C., Veronese, N., Mele, M.L., Rosa-Rizzotto, E., Guido, E., De Lazzari, F., Pilati, P., Farinati, F., Realdon, S. and Solmi, M., 2017. Tumor budding as a risk factor for nodal metastasis in pT1 colorectal cancers: a meta-analysis. *Human pathology, 65*, pp.62-70. |
| 9 | Chen, S., Song, X., Chen, Z., Li, X., Li, M., Liu, H. and Li, J., 2013. CD133 expression and the prognosis of colorectal cancer: a systematic review and meta-analysis. *PloS one, 8*(2), p.e56380. |
| 10 | Chen, Z., He, X., Jia, M., Liu, Y., Qu, D., Wu, D., Wu, P., Ni, C., Zhang, Z., Ye, J. and Xu, J., 2013. β-catenin overexpression in the nucleus predicts progress disease and unfavourable survival in colorectal cancer: a meta-analysis. *PLoS One, 8*(5), p.e63854. |
| 11 | Choi, J.Y., Jung, S., Shim, K.N., Cho, W.Y., Keum, B., Byeon, J.S., Huh, K.C., Jang, B.I., Chang, D.K., Jung, H.Y. and Kong, K., 2015. Meta-analysis of predictive clinicopathologic factors for lymph node metastasis in patients with early colorectal carcinoma. *Journal of Korean Medical Science, 30*(4), pp.398-406. |
| 12 | Glasgow, S.C., Bleier, J.I., Burgart, L.J., Finne, C.O. and Lowry, A.C., 2012. Meta-analysis of histopathological features of primary colorectal cancers that predict lymph node metastases. *Journal of Gastrointestinal Surgery*, *16*(5), pp.1019-1028. |
| 13 | He, X., Chen, Z., Jia, M. and Zhao, X., 2013. Downregulated E-cadherin expression indicates worse prognosis in Asian patients with colorectal cancer: evidence from meta-analysis. *PloS one*, *8*(7), p.e70858. |
| 14 | Huang, X., Shen, W., Xi, H., Zhang, K., Cui, J., Wei, B. and Chen, L., 2016. Prognostic role of extracellular matrix metalloproteinase inducer/CD147 in gastrointestinal cancer: a meta-analysis of related studies. *Oncotarget*, *7*(49), p.81003. |
| 15 | Ichimasa, K., Kudo, S.E., Miyachi, H., Kouyama, Y., Ishida, F., Baba, T., Katagiri, A., Wakamura, K., Hayashi, T., Hisayuki, T. and Kudo, T., 2017. Patient gender as a factor associated with lymph node metastasis in T1 colorectal cancer: a systematic review and meta-analysis. *Molecular and clinical oncology*, *6*(4), pp.517-524. |
| 16 | Kang, H.S., Kwon, M.J., Kim, T.H., Han, J. and Ju, Y.S., 2019. ReviewLymphovascular invasion as a prognostic value in small rectal neuroendocrine tumor treated by local excision: A systematic review and meta-analysis. *Pathology-Research and Practice*, p.152642. |
| 17 | Katsuno, H., Zacharakis, E., Aziz, O., Rao, C., Deeba, S., Paraskeva, P., Ziprin, P., Athanasiou, T. and Darzi, A., 2008. Does the presence of circulating tumor cells in the venous drainage of curative colorectal cancer resections determine prognosis? A meta-analysis. *Annals of surgical oncology, 15*(11), pp.3083-3091. |
| 18 | Li, C., Zuo, D., Yin, L., Lin, Y., Li, C., Liu, T. and Wang, L., 2018. Prognostic Value of MUC2 Expression in Colorectal Cancer: A Systematic Review and Meta-Analysis. *Gastroenterology research and practice*, *2018*. |
| 19 | Li, Y., Wei, J., Xu, C., Zhao, Z. and You, T., 2014. Prognostic significance of cyclin D1 expression in colorectal cancer: a meta-analysis of observational studies. *PloS one*, *9*(4), p.e94508. |
| 20 | Li, Y. and Li, W., 2017. BRAF mutation is associated with poor clinicopathological outcomes in colorectal cancer: a meta-analysis. *Saudi journal of gastroenterology: official journal of the Saudi Gastroenterology Association, 23*(3), p.144. |
| 21 | Mou, S., Soetikno, R., Shimoda, T., Rouse, R. and Kaltenbach, T., 2013. Pathologic predictive factors for lymph node metastasis in submucosal invasive (T1) colorectal cancer: a systematic review and meta-analysis. *Surgical endoscopy*, *27*(8), pp.2692-2703. |
| 22 | Rogers, A.C., Winter, D.C., Heeney, A., Gibbons, D., Lugli, A., Puppa, G. and Sheahan, K., 2016. Systematic review and meta-analysis of the impact of tumour budding in colorectal cancer. *British journal of cancer*, *115*(7), p.831. |
| 23 | Rogers, A.C., Handelman, G.S., Solon, J.G., McNamara, D.A., Deasy, J. and Burke, J.P., 2017. Meta-analysis of the clinicopathological characteristics and peri-operative outcomes of colorectal cancer in obese patients. *Cancer epidemiology, 51*, pp.23-29. |
| 24 | Siddiqui, M.R., Simillis, C., Hunter, C., Chand, M., Bhoday, J., Garant, A., Vuong, T., Artho, G., Rasheed, S., Tekkis, P. and Abulafi, A.M., 2017. A meta-analysis comparing the risk of metastases in patients with rectal cancer and MRI-detected extramural vascular invasion (mrEMVI) vs mrEMVI-negative cases. *British journal of cancer, 116*(12), p.1513. |
| 25 | Wada, H., Shiozawa, M., Katayama, K., Okamoto, N., Miyagi, Y., Rino, Y., Masuda, M. and Akaike, M., 2015. Systematic review and meta-analysis of histopathological predictive factors for lymph node metastasis in T1 colorectal cancer. *Journal of gastroenterology*, *50*(7), pp.727-734. |
| 26 | Wang, H.L., Zhang, Y., Liu, P. and Zhou, P.Y., 2014. RETRACTED ARTICLE: Aberrant promoter methylation of RASSF1A gene may be correlated with colorectal carcinogenesis: a meta-analysis. *Molecular biology reports*, *41*(6), pp.3991-3999. |
| 27 | Wang, K., Xu, J., Zhang, J. and Huang, J., 2012. Prognostic role of CD133 expression in colorectal cancer: a meta-analysis. *BMC cancer*, *12*(1), p.573. |
| 28 | Wang, Y., Yao, X., Ge, J., Hu, F. and Zhao, Y., 2014. Can vascular endothelial growth factor and microvessel density be used as prognostic biomarkers for colorectal cancer? A systematic review and meta-analysis. *The Scientific World Journal*, *2014*. |
| 29 | Wu, S.W., Ma, C.C. and Li, W.H., 2015. Does overexpression of HER-2 correlate with clinicopathological characteristics and prognosis in colorectal cancer? Evidence from a meta-analysis. *Diagnostic pathology*, *10*(1), p.144. |
| 30 | Yang, C., Zou, K., Zheng, L. and Xiong, B., 2017. Prognostic and clinicopathological significance of circulating tumor cells detected by RT-PCR in non-metastatic colorectal cancer: a meta-analysis and systematic review. *BMC cancer*, *17*(1), p.725. |
| 31 | Zhang, Q., Chen, Y., Zhang, B., Shi, B., Weng, W., Chen, Z., Guo, N., Hua, Y. and Zhu, L., 2013. Hypoxia-inducible factor-1α polymorphisms and risk of cancer metastasis: a meta-analysis. *PloS one, 8*(8), p.e70961. |
| 32 | Zhou, N. and Gu, Q., 2018. Prognostic and clinicopathological value of p16 protein aberrant expression in colorectal cancer: A PRISMA-compliant Meta-analysis. *Medicine*, *97*(12). |
| 33 | Zhou, X., Xie, H., Xie, L., Li, J. and Fu, W., 2013. Factors associated with lymph node metastasis in radically resected rectal carcinoids: a systematic review and meta-analysis. *Journal of Gastrointestinal Surgery*, *17*(9), pp.1689-1697. |
| **CRC recurrence and risk factors** | |
| 34 | Doleman, B., Mills, K.T., Lim, S., Zelhart, M.D. and Gagliardi, G., 2016. Body mass index and colorectal cancer prognosis: a systematic review and meta-analysis. *Techniques in coloproctology*, *20*(8), pp.517-535. |
| 35 | Ha, G.W., Kim, J.H. and Lee, M.R., 2017. Oncologic impact of anastomotic leakage following colorectal cancer surgery: a systematic review and meta-analysis. *Annals of surgical oncology*, *24*(11), pp.3289-3299. |
| 36 | Knijn, N., Mogk, S.C., Teerenstra, S., Simmer, F. and Nagtegaal, I.D., 2016. Perineural invasion is a strong prognostic factor in colorectal cancer. *The American journal of surgical pathology*, *40*(1), pp.103-112. |
| 37 | Knijn, N., van Exsel, U.E., de Noo, M.E. and Nagtegaal, I.D., 2018. The value of intramural vascular invasion in colorectal cancer–a systematic review and meta‐analysis. *Histopathology*, *72*(5), pp.721-728. |
| 38 | Kunzmann, A.T., Murray, L.J., Cardwell, C.R., McShane, C.M., McMenamin, Ú.C. and Cantwell, M.M., 2013. PTGS2 (Cyclooxygenase-2) expression and survival among colorectal cancer patients: a systematic review. *Cancer Epidemiology and Prevention Biomarkers*, *22*(9), pp.1490-1497. |
| 39 | Lu, Z.R., Rajendran, N., Lynch, A.C., Heriot, A.G. and Warrier, S.K., 2016. Anastomotic leaks after restorative resections for rectal cancer compromise cancer outcomes and survival. *Diseases of the Colon & Rectum*, *59*(3), pp.236-244. |
| 40 | Mills, K.T., Bellows, C.F., Hoffman, A.E., Kelly, T.N. and Gagliardi, G., 2013. Diabetes and colorectal cancer prognosis: a meta-analysis. *Diseases of the colon and rectum*, *56*(11), p.1304. |
| 41 | Mirnezami, A., Mirnezami, R., Chandrakumaran, K., Sasapu, K., Sagar, P. and Finan, P., 2011. Increased local recurrence and reduced survival from colorectal cancer following anastomotic leak: systematic review and meta-analysis. *Annals of surgery*, *253*(5), pp.890-899. |
| 42 | Rekhraj, S., Aziz, O., Prabhudesai, S., Zacharakis, E., Mohr, F., Athanasiou, T., Darzi, A. and Ziprin, P., 2008. Can intra-operative intraperitoneal free cancer cell detection techniques identify patients at higher recurrence risk following curative colorectal cancer resection: a meta-analysis. *Annals of surgical oncology*, *15*(1), pp.60-68. |
| 22 | Rogers, A.C., Winter, D.C., Heeney, A., Gibbons, D., Lugli, A., Puppa, G. and Sheahan, K., 2016. Systematic review and meta-analysis of the impact of tumour budding in colorectal cancer. *British journal of cancer*, *115*(7), p.831. |
| 24 | Siddiqui, M.R., Simillis, C., Hunter, C., Chand, M., Bhoday, J., Garant, A., Vuong, T., Artho, G., Rasheed, S., Tekkis, P. and Abulafi, A.M., 2017. A meta-analysis comparing the risk of metastases in patients with rectal cancer and MRI-detected extramural vascular invasion (mrEMVI) vs mrEMVI-negative cases. *British journal of cancer, 116*(12), p.1513. |
| 43 | Veronese, N., Nottegar, A., Pea, A., Solmi, M., Stubbs, B., Capelli, P., Sergi, G., Manzato, E., Fassan, M., Wood, L.D. and Scarpa, A., 2015. Prognostic impact and implications of extracapsular lymph node involvement in colorectal cancer: a systematic review with meta-analysis. *Annals of oncology*, *27*(1), pp.42-48. |

Red: common studies for two study outcomes

| **Table S3**: **Quantitative synthesis of all 61 eligible meta-analyses of observational studies investigating the associations between risk factors and colorectal cancer metastasis** | | | | | | | | | | | | | |
| --- | --- | --- | --- | --- | --- | --- | --- | --- | --- | --- | --- | --- | --- |
| **Ref.** | **n of study** | **Study design** | **Population** | **Outcome** | **Risk factor** | **n  (event)** | **N (sample)** | **Metric** | **MA model** | **Effect size (95% CI)** | **P-value** | **I²** | **Assessment tool and risk of bias** |
|  |  |  |  |  |  |  |  |  |  |  |  |  |  |
| **Histopathological risk factor** | | | | | | | | | | | | | |
| Beaton, 2013 (6) | 6 | retrospective | pT1 CRC | lymph node metastasis in pT1 CRC | submucosal invasion > 1mm | 249 | 2077 | OR | random | 3.87 (1.50-10.00) | 0.005 | 54% | NOS (total score: 9); Low risk (3 studies) High risk (3 studies) |
| Mou, 2013 (21) | 3 | retrospective | pT1 CRC | lymph node metastasis in pT1 CRC | submucosal invasion > 1mm | 122 | 993 | RR | random | 5.93 (0.92-38.04) | 0.06 | 57% | NOS (total score: 9); High risk (3 studies) |
| Bosch, 2013 (7) | 5 | 4 retrospective;  1 prospective | pT1 CRC | lymph node metastasis in pT1 CRC | submucosal invasion ≥ 1mm | 182 | 1835 | RR | random | 5.20 (1.80-15.40) | 0.003 | 36% | No |
| Choi, 2015 (11) | 10 | retrospective | pT1 CRC | lymph node metastasis in pT1 CRC | submucosal invasion ≥ 1mm | 332 | 2922 | OR | fixed | 3.00 (1.36-6.62) | 0.007 | 56% | No |
| Bosch, 2013 (7) | 10 | 9 retrospective;  1 case-control | pT1 CRC | lymph node metastasis in pT1 CRC | lymphatic invasion | 233 | 1931 | RR | random | 5.20 (4.00-6.80) | <0.001 | 0% | No |
| Mou, 2013 (21) | 4 | retrospective | pT1 CRC | lymph node metastasis in pT1 CRC | lymphatic invasion | 119 | 976 | RR | random | 4.15 (2.88-5.97) | <0.00001 | 0% | NOS (total score: 9); High risk (4 studies) |
| Choi, 2015 (11) | 12 | retrospective | pT1 CRC | lymph node metastasis in pT1 CRC | lymphatic invasion | 392 | 3347 | OR | fixed | 6.91 (5.40-8.85) | <0.001 | 0% | No |
| Glasgow, 2012 (12)* | 35 | NA | CRC | lymph node metastasis in CRC | lymphatic invasion | NA | NA | OR | fixed | 8.62 (7.55-9.84) | NA | NA | No |
| Kang, 2019 (16) | 3 | retrospective | small rectal NETs | lymph node metastasis in small rectal NETs treated by local excision | lymphatic invasion | 77 | 493 | OR | random | 6.02 (0.71-51.17) | 0.10 | 86% | NOS (total score: 9);  Moderate risk (3 studies) |
| Mou, 2013 (21) | 3 | retrospective | pT1 CRC | lymph node metastasis in pT1 CRC | vascular invasion | 108 | 911 | RR | random | 2.59 (1.82- 3.68) | <0.00001 | 0% | NOS (total score: 9); High risk (3 studies) |
| Choi, 2015 (11) | 5 | retrospective | pT1 CRC | lymph node metastasis in pT1 CRC | vascular invasion | 209 | 1731 | OR | fixed | 2.70 (1.95-3.74) | < 0.001 | 0% | No |
| Glasgow, 2012 (12)* | 23 | NA | CRC | lymph node metastasis in CRC | vascular invasion | NA | NA | OR | fixed | 2.87 (2.39-1.94) | NA | NA | No |
| Zhou, 2013 (33) | 3 | retrospective | rectal cancer | lymph node metastasis in rectal cancer | vascular invasion | 66 | 168 | OR | fixed | 6.26 (2.91-13.42) | <0.00001 | 29% | D.H Checklist (total score: 9); Low risk (3 studies) |
| Kang, 2019 (16) | 2 | retrospective | small rectal NETs | lymph node metastasis in small rectal NETs treated by local excision | vascular invasion | 62 | 211 | OR | random | 3.84 (2.01-7.31) | <0.0001 | 0% | NOS (total score: 9);  Moderate risk (2 studies) |
| Beaton, 2013 (6) | 8 | 7 retrospective;  1 prospective | pT1 CRC | lymph node metastasis in pT1 CRC | lymphovascular invasion | 255 | 1695 | OR | random | 4.81 (3.14-7.37) | <0.00001 | 21% | NOS (total score: 9); Low risk (5 studies) High risk (3 studies) |
| Wada, 2015 (25) | 5 | retrospective | pT1 CRC | lymph node metastasis in pT1 CRC | lymphovascular invasion | 208 | 770 | OR | random | 5.19 (3.31-8.15) | 0.01 | 0% | NOS (total score: 9); Low risk (5 studies) |
| Choi, 2015 (11) | 8 | retrospective | pT1 CRC | lymph node metastasis in pT1 CRC patients who underwent additional surgeries after an endoscopic resection | lymphovascular invasion | 37 | 313 | OR | fixed | 5.47 (2.46-12.17) | <0.001 | 0% | No |
| Kang, 2019 (16) | 5 | retrospective | small rectal NETs | lymph node metastasis in small rectal NETs treated by local excision | lymphovascular invasion | 84 | 517 | OR | random | 4.98 (1.13-21.95) | 0.03 | 73% | NOS (total score: 9);  Moderate risk (4 studies)  High risk (1 study) |
| Beaton, 2013 (6) | 7 | 6 retrospective;  1 prospective | pT1 CRC | lymph node metastasis in pT1 CRC | tumour budding | 144 | 720 | OR | random | 7.74 (4.47-13.39) | <0.001 | 18% | NOS (total score: 9); Low risk (1 study) High risk (6 studies) |
| Bosch, 2013 (7) | 7 | 5 retrospective;  1 prospective;  1 case-control | pT1 CRC | lymph node metastasis in pT1 CRC | tumour budding | 233 | 1991 | RR | random | 5.10 (3.60-6.96) | <0.001 | 20% | No |
| Cappellesso, 2017 (8) | 41 | 39 retrospective;  2 case-control | pT1 CRC | lymph node metastasis in pT1 CRC | tumour budding | 1240 | 10128 | OR | random | 6.44 (5.26-7.87) | <0.001 | 30% | STROBE; NA |
| Mou, 2013 (21) | 2 | retrospective | pT1 CRC | lymph node metastasis in pT1 CRC | tumour budding | 97 | 846 | RR | random | 3.26 (2.17- 4.90) | <0.00001 | 0% | NOS (total score: 9); High risk (2 studies) |
| Wada, 2015 (25) | 4 | retrospective | pT1 CRC | lymph node metastasis in pT1 CRC | tumour budding | 79 | 634 | OR | random | 7.45 (4.27-13.02) | 0.0077 | 0% | NOS (total score: 9); Low risk (4 studies) |
| Choi, 2015 (11) | 7 | retrospective | pT1 CRC | lymph node metastasis in pT1 CRC | tumour budding | 257 | 2119 | OR | fixed | 4.59 (3.44 - 6.13) | < 0.001 | 42% | No |
| Glasgow, 2012 (12)* | 26 | NA | CRC | lymph node metastasis in CRC | tumour budding | NA | NA | OR | fixed | 5.75 (5.05-6.56) | NA | NA | No |
| Rogers, 2016 (22) | 25 | 24 retrospective;  1 case-control | CRC | lymph node metastasis in CRC | tumour budding | 1808 | 6739 | OR | random | 4.94 (3.96-6.17) | <0.00001 | 53% | NOS (total score: 9); Low risk (24 studies) High risk (1 study) |
| Beaton, 2013 (6) | 13 | 12 retrospective;  1 prospective | pT1 CRC | lymph node metastasis in pT1 CRC | poor differentiation | 332 | 2722 | OR | random | 5.60 (2.90-10.82) | <0.00001 | 31% | NOS (total score: 9); Low risk (5 studies) High risk (8 studies) |
| Bosch, 2013 (7) | 13 | 11 retrospective;  2 prospective | pT1 CRC | lymph node metastasis in pT1 CRC | poor differentiation | 295 | 2847 | RR | random | 4.80 (3.30-6.90) | <0.001 | 25% | No |
| Mou, 2013 (21) | 2 | retrospective | pT1 CRC | lymph node metastasis in pT1 CRC | poor differentiation | 16 | 111 | RR | random | 7.17 (3.70- 13.91) | <0.00001 | 0% | NOS (total score: 9); High risk (2 studies) |
| Choi, 2015 (11) | 7 | retrospective | pT1 CRC | lymph node metastasis in pT1 CRC | poor or moderate differentiation | 273 | 1628 | OR | fixed | 5.27 (3.66 - 7.58) | < 0.001 | 0% | No |
| Choi, 2015 (11) | 3 | retrospective | pT1 CRC | lymph node metastasis in pT1 CRC patients who underwent additional surgeries after an endoscopic resection | poor or moderate differentiation | 16 | 209 | OR | fixed | 4.07 (1.08-15.33) | 0.04 | 8% | No |
| Glasgow, 2012 (12)* | 29 | NA | CRC | lymph node metastasis in CRC | overall differentiation | NA | NA | OR | fixed | 2.38 (2.32-2.44) | NA | NA | No |
| Glasgow, 2012 (12)* | 25 | NA | CRC | lymph node metastasis in CRC | tumour stage (T2 vs. T1) | NA | NA | OR | fixed | 2.62 (2.46-2.79) | NA | NA | No |
| Zhou, 2013 (33) | 7 | retrospective | rectal cancer | lymph node metastasis in rectal cancer | tumour size>1 cm | 140 | 348 | OR | fixed | 7.36 (4.07-13.31) | <0.00001 | 25% | D.H Checklist (total score: 9); Low risk (7 studies) |
| Zhou, 2013 (33) | 2 | retrospective | rectal cancer | lymph node metastasis in rectal cancer | central depression | 33 | 76 | OR | fixed | 3.00 (1.07-8.43) | 0.04 | 0% | D.H Checklist (total score: 9); Low risk (2 studies) |
| Zhou, 2013 (33) | 6 | retrospective | rectal cancer | lymph node metastasis in rectal cancer | muscularis properia invasion | 127 | 322 | OR | fixed | 5.62 (3.08-10.25) | <0.00001 | 27% | D.H Checklist (total score: 9); Low risk (6 studies) |
| Siddiqui, 2017 (24) | 3 | retrospective | rectal cancer | synchronous metastasis in rectal cancer | MRI-detected extramural vascular invasion (mrEMVI) | 122 | 804 | OR | fixed | 5.68 (3.75-8.61) | <0.001 | 0% | SIGN;  level 3: 3 studies |
| **Biomarker** | | | | | | | | | | | | | |
| He, 2013 (13) | 14 | retrospective | CRC | lymph node metastasis in CRC | downregulated E-cadherin expression | 658+NA | 1593 | OR | random | 0.49 (0.32-0.74) | 0.001 | 59% | NOS (total score: 9); Low risk (11 studies) High risk (2 studies) |
| He, 2013 (13) | 8 | retrospective | CRC | distant metastasis in CRC | downregulated E-cadherin expression | 136+NA | 983 | OR | random | 0.45 (0.22-0.91) | 0.025 | 77.6% | NOS (total score: 9); Low risk (6 studies) High risk (2 studies) |
| Huang, 2016 (14) | 3 | retrospective | CRC | lymph node metastasis in CRC | CD147 expression | 374 | 815 | OR | random | 1.41 (0.76-2.59) | <0.05 | 74.4% | ELCWP scale; NA |
| Huang, 2016 (14) | 2 | retrospective | CRC | distant metastasis in CRC | CD147 expression | 56 | 538 | OR | random | 2.32 (0.24-22.17) | <0.05 | 87% | ELCWP scale; NA |
| Yang, 2017 (30) | 7 | prospective | CRC | lymph node metastasis in CRC | circulating tumour cells | 721+NA | 1535 | RR | random | 1.62 (1.17-2.23) | 0.003 | 74.6% | NOS (total score: 9); Low risk (3 studies) High risk (4 studies) |
| Katsuno, 2008 (17) | 5 | prospective | CRC | hepatic metastasis (distant) in CRC | circulating tumour cells | 38 | 310 | OR | random | 6.38 (2.67-15.25) | <0.0001 | 0% | No |
| Wang, 2014 (28)* | 15 | NA | CRC | lymph node metastasis in CRC | VEGF expression | NA | NA | OR | random | 2.51 (1.51-4.15) | < 0.01 | NA | No |
| Wang, 2014 (28)* | 9 | NA | CRC | vascular metastasis in CRC | VEGF expression | NA | NA | OR | random | 2.38 (1.49-3.79) | < 0.01 | NA | No |
| Wang, 2014 (28)* | 8 | NA | CRC | distant metastasis in CRC | VEGF expression | NA | NA | OR | random | 4.22 (2.93-6.06) | < 0.01 | NA | No |
| Wang, 2014 (28)* | 8 | NA | CRC | lymph node metastasis in CRC | MVD expression | NA | NA | OR | random | 1.84 (1.19-2.85) | < 0.01 | NA | No |
| Wang, 2014 (28)* | 7 | NA | CRC | vascular metastasis in CRC | MVD expression | NA | NA | OR | random | 1.43 (1.06-1.92) | 0.02 | NA | No |
| Wang, 2012 (27) | 7 | retrospective | CRC | lymph node metastasis in CRC | CD133 expression | 751 | 1629 | OR | fixed | 1.16 (0.87-1.54) | 0.315 | 19.5% | No |
| Chen, 2013 (9) | 4 | retrospective | CRC | distant metastasis in CRC | CD133 expression | 95 | 1064 | RR | fixed | 1.42 (0.92-2.19) | 0.11 | 43.9% | No |
| Wu, 2015 (29) | 8 | retrospective | CRC | lymph node metastasis in CRC | HER-2 immunohistochemical expression | 369 | 1289 | OR | random | 1.90 (0.90-4.02) | 0.09 | 82% | No |
| Zhou, 2018 (32) | 14 | case-control | CRC | lymph node metastasis in CRC | p16 protein expression | 321+NA | 800+NA | OR | random | 0.52 (0.32-0.86) | <0.05 | 54.8% | NOS (total score: 9); Low risk (6 studies) NA risk (8 studies) |
| Li, 2018 (18) | 8 | retrospective | CRC | lymph node metastasis in CRC | low MUC2 expression level | 592 | 1335 | RR | random | 1.41 (1.25-1.60) | <0.00001 | 49% | NOS (total score: 9); Low risk (8 studies) |
| Li, 2014 (19) | 9 | 6 prospective; 3 case-control | CRC | distant metastasis in CRC | cyclin D1 overexpression | 88+NA | 1515 | OR | random | 0.60 (0.36-0.99) | 0.047 | 65.3% | NOS (total score: 9); Low risk (9 studies) |
| Chen, 2013 (10) | 5 | retrospective | CRC | distant metastasis in CRC | ℬ-catenin overexpression in the nucleus | 217 | 628 | OR | random | 0.49 (0.25-0.96) | 0.039 | 65.1% | NOS (total score: 9); Low risk (4 studies) High risk (1 study) |
| **Genetic risk factor** | | | | | | | | | | | | | |
| Zhang, 2013 (31)* | 2 | case-control | CRC | CRC metastasis | HIF-1a C1772 T | 245 | 398 | OR | fixed | 1.23 (0.74-2.07) | 0.43 | 0% | No |
| Li, 2017 (20) | 4 | retrospective | CRC | lymph node metastasis in CRC | BRAF mutation | 100 | 1142 | OR | fixed | 0.74 (0.47-1.17) | 0.20 | 0% | No |
| Wang, 2014 (26) | 3 | retrospective | CRC | lymph node metastasis in CRC | RASSF1A promoter methylation | 91 | 184 | OR | random | 1.65 (0.87-3.14) | 0.127 | 63.4% | NOS (total score: 9);  Low risk (2 studies)  Moderate risk (1 study) |
| Wang, 2014 (26) | 4 | retrospective | CRC | distant metastasis in CRC | RASSF1A promoter methylation | 173 | 417 | OR | fixed | 2.59 (1.46-4.60) | 0.037 | 37.9% | NOS (total score: 9);  Low risk (3 studies)  Moderate risk (1 study) |
| **Demographic risk factor** | | | | | | | | | | | | | |
| Ichimasa, 2017 (15) | 4 | retrospective | pT1 CRC | lymph node metastasis in pT1 CRC | female gender | 113 | 1329 | RR | random | 2.45 (1.03-3.88) | <0.05 | 90.1% | GRADE;  Low (+OOO) risk (4 studies) |
| **Anthropometric indices** | | | | | | | | | | | | | |
| Rogers, 2017 (23)* | 11 | retrospective | CRC | lymph node metastasis in CRC | obese | NA | 33003 | OR | fixed | 1.20 (1.10-1.20) | <0.00001 | 37% | MINOR score (total score: 24);  Low risk (11 studies) |

*Meta-analysis did not provide detailed forest plot (summary effect size)

Abbreviation: OR, odds ratio; RR, risk ratio; CI, confidence interval; CRC, colorectal cancer; NET, neuroendocrine tumour; MINORS, Methodological index for non-randomized studies; NOS, Newcastle Ottawa Scale (>=6: low risk, <6: high risk); D.H Checklist, Duckitt and Harrington Checklist; ELCWP scale, European Lung Cancer Working Party; GRADE, The Grading of Recommendations Assessment, Development and Evaluation; SIGN, Scottish Intercollegiate Guidelines Network; STROBE, Strengthening the Reporting of Observational studies in Epidemiology

| **Table S4: Quantitative synthesis of all 20 eligible meta-analyses of observational studies investigating the associations between risk factors and colorectal cancer recurrence** | | | | | | | | | | | | | |  |
| --- | --- | --- | --- | --- | --- | --- | --- | --- | --- | --- | --- | --- | --- | --- |
| **Ref.** | **n of study** | **Study design** | **Population** | **Outcome** | **Risk factor** | **n  (event)** | **N (sample)** | **Metric** | **MA model** | **Effect size (95% CI)** | **P-value** | **I²** | **Assessment tool and risk of bias** | |
|  |  |  |  |  |  |  |  |  |  |  |  |  |  |  |
| **Histopathological risk factor** | | | | | | | | | | | | | |  |
| Knijn, 2018 (37) | 2 | 1 prospective;  1 case-control | CRC | local recurrence in CRC | intramural vascular invasion (IMVI) | 96 | 503 | RR | random | 1.50 (0.98-2.30) | 0.06 | 0% | REMARK  Low risk (1 study: 88.9% items reported); Moderate risk (1 study:55% items reported) | |
| Rogers, 2016 (22) | 12 | 11 retrospective;  1 case-control | CRC | overall recurrence in CRC | tumour budding | 551 | 2773 | OR | random | 5.50 (3.64-8.29) | <0.00001 | 61% | NOS (total score: 9); Low risk (12 studies) | |
| Veronese, 2015 (43) | 9 | prospective | CRC | overall recurrence in CRC | extranodal extension (ENE) | 389 | 877 | RR | random | 2.07 (1.65-2.61) | <0.0001 | 47% | NOS (total score: 9); Low risk (9 studies) | |
| Knijn, 2016 (36) | 5 | retrospective | rectal cancer | local recurrence in rectal cancer | perineural invasion (PNI) | 146 | 1700 | RR | random | 3.22 (2.33-4.44) | <0.00001 | 22% | REMARK  Low risk (2 studies: 83.3% items reported); Moderate risk (3 studies:61.1%, 50% items reported) | |
| Siddiqui, 2017 (24) | 6 | retrospective | rectal cancer | distant metastatic recurrence in rectal cancer | MRI-detected extramural vascular invasion (mrEMVI) | 284 | 1262 | OR | fixed | 4.02 (2.99-5.39) | <0.001 | 41% | SIGN; level 3 (6 studies) | |
| **Biomarker** | | | | | | | | | | | | | |  |
| Rekhraj, 2008 (42) | 6 | 5 retrospective;  1 prospective | CRC | overall recurrence in CRC | absence of peritoneal free tumour cells in pre-resection | 164 | 593 | OR | random | 0.41 (0.19-0.88) | 0.02 | 32.4% | No | |
| Rekhraj, 2008 (42) | 3 | 2 retrospective;  1 prospective | CRC | overall recurrence in CRC | absence of peritoneal free tumour cells in post-resection | 57 | 252 | OR | random | 0.07 (0.03-0.18) | <0.00001 | 0% | No | |
| **Genetic risk factor** | | | | | | | | | | | | | | |
| Kunzmann, 2013(38) | 8 | retrospective | CRC | overall recurrence in CRC | PTGS2 (also known as COX-2) | 232+NA | 1516 | HR | random | 2.79 (1.76-4.41) | < 0.001 | 36% | No | |
| **Clinical risk factor** | | | | | | | | | | | | | |  |
| Ha, 2017 (35) | 26 | 12 retrospective;  14 prospective | CRC | local recurrence in CRC | anastomotic leakage (AL) | 3675 | 39745 | RR | random | 1.90 (1.48-2.44) | <0.00001 | 78% | QUIPS;  NA | |
| Mirnezami, 2011 (41) | 9 | 5 retrospective;  4 prospective | CRC | local recurrence in CRC | anastomotic leakage (AL) | 748 | 7136 | OR | random | 2.90 (1.78-4.71) | 0.0001 | 69.9% | SIGN; level 3 (9 studies) | |
| Ha, 2017 (35) | 11 | 5 retrospective;  6 prospective | CRC | distant recurrence in CRC | anastomotic leakage (AL) | 2086 | 10392 | RR | random | 1.20 (0.94-1.53) | 0.15 | 61% | QUIPS;  NA | |
| Mirnezami, 2011 (41) | 7 | 4 retrospective;  3 prospective | CRC | distant recurrence in CRC | anastomotic leakage (AL) | 1517 | 7580 | OR | random | 1.38 (0.96-1.99) | 0.083 | 62.5% | SIGN; level 3 (7 studies) | |
| Lu, 2016 (39) | 11 | 6 retrospective;  5 prospective | rectal cancer | local recurrence in rectal cancer | anastomotic leakage (AL) | 1199 | 13665 | OR | random | 1.61 (1.25-2.09) | < 0.001 | 37.4% | NOS (total score: 9); Low risk (11 studies) | |
| Mirnezami, 2011 (41) | 13 | 5 retrospective;  8 prospective | rectal cancer | local recurrence in rectal cancer | anastomotic leakage (AL) | 1256 | 12202 | OR | random | 2.05 (1.51-2.80) | 0.0001 | 61.2% | SIGN; level 3 (13 studies) | |
| Lu, 2016 (39) | 4 | 1 retrospective;  3 prospective | rectal cancer | distant recurrence in rectal cancer | anastomotic leakage (AL) | 1163 | 5221 | OR | random | 1.07 (0.87-1.33) | 0.52 | 0% | NOS (total score: 9); Low risk (4 studies) | |
| Mirnezami, 2011 (41) | 3 | prospective | colon cancer | local recurrence in colon cancer | anastomotic leakage (AL) | 133 | 1990 | OR | random | 2.16 (0.88-5.29) | 0.094 | 0% | SIGN;  level 3 (3 studies) | |
| **Comorbidity** | | | | | | | | | | | | | |  |
| Mills, 2013 (40) | 3 | 2 retrospective;  3 prospective | CRC | overall recurrence in CRC | diabetes | 1678 | 4979 | RR | random | 1.24 (0.99-1.55) | >0.05 | 63.4% | STROBE (16 item); Moderate risk (2 studies: 5-6 items reported); High risk (1 study: 4 items reported) | |
| **Anthropometric indices** | | | | | | | | | | | | | |  |
| Doleman, 2016 (34) | 6 | 5 retrospective;  1 prospective | CRC | overall recurrence in CRC | underweight | 1004+NA | 37223 | RR | random | 1.13 (1.05-1.21) | <0.05 | 0% | STROBE (16 item); Moderate risk (6 studies: 6 items reported) | |
| Doleman, 2016 (34) | 6 | 5 retrospective;  1 prospective | CRC | overall recurrence in CRC | overweight | 1617+NA | 37223 | RR | random | 1.00 (0.96-1.05) | >0.05 | 0% | STROBE (16 item); Moderate risk (6 studies: 6 items reported) | |
| Doleman, 2016 (34) | 6 | 5 retrospective;  1 prospective | CRC | overall recurrence in CRC | obese | 1139+NA | 37223 | RR | random | 1.07 (1.02-1.13) | <0.05 | 0% | STROBE (16 item); Moderate risk (6 studies: 6 items reported) | |

Abbreviation: OR, odds ratio; RR, risk ratio; CI, confidence interval; CRC, colorectal cancer; NOS, Newcastle Ottawa Scale (>=6: low risk, <6: high risk); SIGN, Scottish Intercollegiate Guidelines Network; STROBE, Strengthening the Reporting of Observational studies in Epidemiology; REMARK, Reporting recommendations for tumour Marker prognostic studies; QUIPS, Quality In Prognosis Studies

| **Table S5: Overlapping meta-analyses of observational studies investigating the associations between the same risk factor and the same outcome** | | | | | |
| --- | --- | --- | --- | --- | --- |
| **Population** | **Outcome** | **Risk factor** | **No. overlapping meta-analyses** | **Agreement of direction of point estimate** | **Agreement of presence of nominal significance (p<0.05)** |
| **Risk factors and CRC metastasis** | | | | | |
| pT1 CRC | lymph node metastasis in pT1 CRC | submucosal invasion (>= 1mm) | 4 | Y | N |
| pT1 CRC | lymph node metastasis in pT1 CRC | lymphatic invasion | 3 | Y | Y |
| pT1 CRC | lymph node metastasis in pT1 CRC | vascular invasion | 2 | Y | Y |
| pT1 CRC | lymph node metastasis in pT1 CRC | lymphovascular invasion | 2 | Y | Y |
| pT1 CRC | lymph node metastasis in pT1 CRC | tumour budding | 6 | Y | Y |
| CRC | lymph node metastasis in CRC | tumour budding | 2 | Y | Y |
| pT1 CRC | lymph node metastasis in pT1 CRC | tumour differentiation | 4 | Y | Y |
| **Risk factors and CRC recurrence** | | | | | |
| CRC | local recurrence in CRC | anastomotic leakage (AL) | 2 | Y | Y |
| CRC | distant recurrence in CRC | anastomotic leakage (AL) | 2 | Y | N |
| rectal cancer | local recurrence in rectal cancer | anastomotic leakage (AL) | 2 | Y | Y |

| **Table S6: Quantitative synthesis of 34 unique meta-analyses of observational studies investigating the associations between risk factors and colorectal cancer metastasis** | | | | | | | | | | | | | |
| --- | --- | --- | --- | --- | --- | --- | --- | --- | --- | --- | --- | --- | --- |
| **Ref.** | **n of study** | **Study design** | **Population** | **Outcome** | **Risk factor** | **n  (event)** | **N (sample)** | **Metric** | **MA model** | **Effect size (95% CI)** | **P-value** | **I²** | **Assessment tool and risk of bias** |
|  |  |  |  |  |  |  |  |  |  |  |  |  |  |
| **Histopathological risk factor** | | | | | | | | | | | | | |
| Choi, 2015 (11) | 10 | retrospective | pT1 CRC | lymph node metastasis in pT1 CRC | submucosal invasion ≥ 1mm | 332 | 2922 | OR | fixed | 3.00 (1.36-6.62) | 0.007 | 56% | No |
| Choi, 2015 (11) | 12 | retrospective | pT1 CRC | lymph node metastasis in pT1 CRC | lymphatic invasion | 392 | 3347 | OR | fixed | 6.91 (5.40-8.85) | <0.001 | 0% | No |
| Kang, 2019 (16) | 3 | retrospective | small rectal NETs | lymph node metastasis in small rectal NETs treated by local excision | lymphatic invasion | 77 | 493 | OR | random | 6.02 (0.71-51.17) | 0.10 | 86% | NOS (total score: 9);  Moderate risk (3 studies) |
| Choi, 2015 (11) | 5 | retrospective | pT1 CRC | lymph node metastasis in pT1 CRC | vascular invasion | 209 | 1731 | OR | fixed | 2.70 (1.95-3.74) | < 0.001 | 0% | No |
| Zhou, 2013 (33) | 3 | retrospective | rectal cancer | lymph node metastasis in rectal cancer | vascular invasion | 66 | 168 | OR | fixed | 6.26 (2.91-13.42) | <0.00001 | 29% | D.H Checklist (total score: 9); Low risk (3 studies) |
| Kang, 2019 (16) | 2 | retrospective | small rectal NETs | lymph node metastasis in small rectal NETs treated by local excision | vascular invasion | 62 | 211 | OR | random | 3.84 (2.01-7.31) | <0.0001 | 0% | NOS (total score: 9);  Moderate risk (2 studies) |
| Beaton, 2013 (6) | 8 | 7 retrospective;  1 prospective | pT1 CRC | lymph node metastasis in pT1 CRC | lymphovascular invasion | 255 | 1695 | OR | random | 4.81 (3.14-7.37) | <0.00001 | 21% | NOS (total score: 9); Low risk (5 studies) High risk (3 studies) |
| Choi, 2015 (11) | 8 | retrospective | pT1 CRC | lymph node metastasis in pT1 CRC patients who underwent additional surgeries after an endoscopic resection | lymphovascular invasion | 37 | 313 | OR | fixed | 5.47 (2.46-12.17) | <0.001 | 0% | No |
| Kang, 2019 (16) | 5 | retrospective | small rectal NETs | lymph node metastasis in small rectal NETs treated by local excision | lymphovascular invasion | 84 | 517 | OR | random | 4.98 (1.13-21.95) | 0.03 | 73% | NOS (total score: 9);  Moderate risk (4 studies)  High risk (1 study) |
| Cappellesso, 2017 (8) | 41 | 39 retrospective;  2 case-control | pT1 CRC | lymph node metastasis in pT1 CRC | tumour budding | 1240 | 10128 | OR | random | 6.44 (5.26-7.87) | <0.001 | 30% | STROBE; NA |
| Rogers, 2016 (22) | 25 | 24 retrospective;  1 case-control | CRC | lymph node metastasis in CRC | tumour budding | 1808 | 6739 | OR | random | 4.94 (3.96-6.17) | <0.00001 | 53% | NOS (total score: 9); Low risk (24 studies) High risk (1 study) |
| Beaton, 2013 (6) | 13 | 12 retrospective;  1 prospective | pT1 CRC | lymph node metastasis in pT1 CRC | poor differentiation | 332 | 2722 | OR | random | 5.60 (2.90-10.82) | <0.00001 | 31% | NOS (total score: 9); Low risk (5 studies) High risk (8 studies) |
| Choi, 2015 (11) | 3 | retrospective | pT1 CRC | lymph node metastasis in pT1 CRC patients who underwent additional surgeries after an endoscopic resection | poor or moderate differentiation | 16 | 209 | OR | fixed | 4.07 (1.08-15.33) | 0.04 | 8% | No |
| Zhou, 2013 (33) | 7 | retrospective | rectal cancer | lymph node metastasis in rectal cancer | tumour size>1 cm | 140 | 348 | OR | fixed | 7.36 (4.07-13.31) | <0.00001 | 25% | D.H Checklist (total score: 9); Low risk (7 studies) |
| Zhou, 2013 (33) | 2 | retrospective | rectal cancer | lymph node metastasis in rectal cancer | central depression | 33 | 76 | OR | fixed | 3.00 (1.07-8.43) | 0.04 | 0% | D.H Checklist (total score: 9); Low risk (2 studies) |
| Zhou, 2013 (33) | 6 | retrospective | rectal cancer | lymph node metastasis in rectal cancer | muscularis properia invasion | 127 | 322 | OR | fixed | 5.62 (3.08-10.25) | <0.00001 | 27% | D.H Checklist (total score: 9); Low risk (6 studies) |
| Siddiqui, 2017 (24) | 3 | retrospective | rectal cancer | synchronous metastasis in rectal cancer | MRI-detected extramural vascular invasion (mrEMVI) | 122 | 804 | OR | fixed | 5.68 (3.75-8.61) | <0.001 | 0% | SIGN;  level 3: 3 studies |
| **Biomarker** | | | | | | | | | | | | | |
| He, 2013 (13) | 14 | retrospective | CRC | lymph node metastasis in CRC | downregulated E-cadherin expression | 658+NA | 1593 | OR | random | 0.49 (0.32-0.74) | 0.001 | 59% | NOS (total score: 9); Low risk (11 studies) High risk (2 studies) |
| He, 2013 (13) | 8 | retrospective | CRC | distant metastasis in CRC | downregulated E-cadherin expression | 136+NA | 983 | OR | random | 0.45 (0.22-0.91) | 0.025 | 77.6% | NOS (total score: 9); Low risk (6 studies) High risk (2 studies) |
| Huang, 2016 (14) | 3 | retrospective | CRC | lymph node metastasis in CRC | CD147 expression | 374 | 815 | OR | random | 1.41 (0.76-2.59) | <0.05 | 74.4% | ELCWP scale; NA |
| Huang, 2016 (14) | 2 | retrospective | CRC | distant metastasis in CRC | CD147 expression | 56 | 538 | OR | random | 2.32 (0.24-22.17) | <0.05 | 87% | ELCWP scale; NA |
| Yang, 2017 (30) | 7 | prospective | CRC | lymph node metastasis in CRC | circulating tumour cells | 721+NA | 1535 | RR | random | 1.62 (1.17-2.23) | 0.003 | 74.6% | NOS (total score: 9); Low risk (3 studies) High risk (4 studies) |
| Katsuno, 2008 (17) | 5 | prospective | CRC | hepatic metastasis (distant) in CRC | circulating tumour cells | 38 | 310 | OR | random | 6.38 (2.67-15.25) | <0.0001 | 0% | No |
| Wang, 2012 (27) | 7 | retrospective | CRC | lymph node metastasis in CRC | CD133 expression | 751 | 1629 | OR | fixed | 1.16 (0.87-1.54) | 0.315 | 19.5% | No |
| Chen, 2013 (9) | 4 | retrospective | CRC | distant metastasis in CRC | CD133 expression | 95 | 1064 | RR | fixed | 1.42 (0.92-2.19) | 0.11 | 43.9% | No |
| Wu, 2015 (29) | 8 | retrospective | CRC | lymph node metastasis in CRC | HER-2 immunohistochemical expression | 369 | 1289 | OR | random | 1.90 (0.90-4.02) | 0.09 | 82% | No |
| Zhou, 2018 (32) | 14 | case-control | CRC | lymph node metastasis in CRC | p16 protein expression | 321+NA | 800+NA | OR | random | 0.52 (0.32-0.86) | <0.05 | 54.8% | NOS (total score: 9); Low risk (6 studies) NA risk (8 studies) |
| Li, 2018 (18) | 8 | retrospective | CRC | lymph node metastasis in CRC | low MUC2 expression level | 592 | 1335 | RR | random | 1.41 (1.25-1.60) | <0.00001 | 49% | NOS (total score: 9); Low risk (8 studies) |
| Li, 2014 (19) | 9 | 6 prospective; 3 case-control | CRC | distant metastasis in CRC | cyclin D1 overexpression | 88+NA | 1515 | OR | random | 0.60 (0.36-0.99) | 0.047 | 65.3% | NOS (total score: 9); Low risk (9 studies) |
| Chen, 2013 (10) | 5 | retrospective | CRC | distant metastasis in CRC | ℬ-catenin overexpression in the nucleus | 217 | 628 | OR | random | 0.49 (0.25-0.96) | 0.039 | 65.1% | NOS (total score: 9); Low risk (4 studies) High risk (1 study) |
| **Genetic risk factor** | | | | | | | | | | | | | |
| Li, 2017 (20) | 4 | retrospective | CRC | lymph node metastasis in CRC | BRAF mutation | 100 | 1142 | OR | fixed | 0.74 (0.47-1.17) | 0.20 | 0% | No |
| Wang, 2014 (26) | 3 | retrospective | CRC | lymph node metastasis in CRC | RASSF1A promoter methylation | 91 | 184 | OR | random | 1.65 (0.87-3.14) | 0.127 | 63.4% | NOS (total score: 9);  Low risk (2 studies)  Moderate risk (1 study) |
| Wang, 2014 (26) | 4 | retrospective | CRC | distant metastasis in CRC | RASSF1A promoter methylation | 173 | 417 | OR | fixed | 2.59 (1.46-4.60) | 0.037 | 37.9% | NOS (total score: 9);  Low risk (3 studies)  Moderate risk (1 study) |
| **Demographic risk factor** | | | | | | | | | | | | | |
| Ichimasa, 2017 (15) | 4 | retrospective | pT1 CRC | lymph node metastasis in pT1 CRC | female gender | 113 | 1329 | RR | random | 2.45 (1.03-3.88) | <0.05 | 90.1% | GRADE;  Low (+OOO) risk (4 studies) |

Abbreviation: OR, odds ratio; RR, risk ratio; CI, confidence interval; CRC, colorectal cancer; NET, neuroendocrine tumour; MINORS, Methodological index for non-randomized studies; NOS, Newcastle Ottawa Scale (>=6: low risk, <6: high risk); D.H Checklist, Duckitt and Harrington Checklist; ELCWP scale, European Lung Cancer Working Party; GRADE, The Grading of Recommendations Assessment, Development and Evaluation; SIGN, Scottish Intercollegiate Guidelines Network; STROBE, Strengthening the Reporting of Observational studies in Epidemiology

| **Table S7: Quantitative synthesis of 17 unique meta-analyses of observational studies investigating the associations between risk factors and colorectal cancer recurrence** | | | | | | | | | | | | | |  |
| --- | --- | --- | --- | --- | --- | --- | --- | --- | --- | --- | --- | --- | --- | --- |
| **Ref.** | **n of study** | **Study design** | **Population** | **Outcome** | **Risk factor** | **n  (event)** | **N (sample)** | **Metric** | **MA model** | **Effect size (95% CI)** | **P-value** | **I²** | **Assessment tool and risk of bias** | |
|  |  |  |  |  |  |  |  |  |  |  |  |  |  |  |
| **Histopathological risk factor** | | | | | | | | | | | | | |  |
| Knijn, 2018 (37) | 2 | 1 prospective;  1 case-control | CRC | local recurrence in CRC | intramural vascular invasion (IMVI) | 96 | 503 | RR | random | 1.50 (0.98-2.30) | 0.06 | 0% | REMARK  Low risk (1 study: 88.9% items reported); Moderate risk (1 study:55% items reported) | |
| Rogers, 2016 (22) | 12 | 11 retrospective;  1 case-control | CRC | overall recurrence in CRC | tumour budding | 551 | 2773 | OR | random | 5.50 (3.64-8.29) | <0.00001 | 61% | NOS (total score: 9); Low risk (12 studies) | |
| Veronese, 2015 (43) | 9 | prospective | CRC | overall recurrence in CRC | extranodal extension (ENE) | 389 | 877 | RR | random | 2.07 (1.65-2.61) | <0.0001 | 47% | NOS (total score: 9); Low risk (9 studies) | |
| Knijn, 2016 (36) | 5 | retrospective | rectal cancer | local recurrence in rectal cancer | perineural invasion (PNI) | 146 | 1700 | RR | random | 3.22 (2.33-4.44) | <0.00001 | 22% | REMARK  Low risk (2 studies: 83.3% items reported); Moderate risk (3 studies:61.1%, 50% items reported) | |
| Siddiqui, 2017 (24) | 6 | retrospective | rectal cancer | distant metastatic recurrence in rectal cancer | MRI-detected extramural vascular invasion (mrEMVI) | 284 | 1262 | OR | fixed | 4.02 (2.99-5.39) | <0.001 | 41% | SIGN; level 3 (6 studies) | |
| **Biomarker** | | | | | | | | | | | | | |  |
| Rekhraj, 2008 (42) | 6 | 5 retrospective;  1 prospective | CRC | overall recurrence in CRC | absence of peritoneal free tumour cells in pre-resection | 164 | 593 | OR | random | 0.41 (0.19-0.88) | 0.02 | 32.4% | No | |
| Rekhraj, 2008 (42) | 3 | 2 retrospective;  1 prospective | CRC | overall recurrence in CRC | absence of peritoneal free tumour cells in post-resection | 57 | 252 | OR | random | 0.07 (0.03-0.18) | <0.00001 | 0% | No | |
| **Genetic risk factor** | | | | | | | | | | | | | | |
| Kunzmann, 2013 (38) | 8 | retrospective | CRC | overall recurrence in CRC | PTGS2 (also known as COX-2) | 232+NA | 1516 | HR | random | 2.79 (1.76-4.41) | < 0.001 | 36% | No | |
| **Clinical risk factor** | | | | | | | | | | | | | |  |
| Ha, 2017 (35) | 26 | 12 retrospective;  14 prospective | CRC | local recurrence in CRC | anastomotic leakage (AL) | 3675 | 39745 | RR | random | 1.90 (1.48-2.44) | <0.00001 | 78% | QUIPS;  NA | |
| Ha, 2017 (35) | 11 | 5 retrospective;  6 prospective | CRC | distant recurrence in CRC | anastomotic leakage (AL) | 2086 | 10392 | RR | random | 1.20 (0.94-1.53) | 0.15 | 61% | QUIPS;  NA | |
| Lu, 2016 (39) | 11 | 6 retrospective;  5 prospective | rectal cancer | local recurrence in rectal cancer | anastomotic leakage (AL) | 1199 | 13665 | OR | random | 1.61 (1.25-2.09) | < 0.001 | 37.4% | NOS (total score: 9); Low risk (11 studies) | |
| Lu, 2016 (39) | 4 | 1 retrospective;  3 prospective | rectal cancer | distant recurrence in rectal cancer | anastomotic leakage (AL) | 1163 | 5221 | OR | random | 1.07 (0.87-1.33) | 0.52 | 0% | NOS (total score: 9); Low risk (4 studies) | |
| Mirnezami, 2011 (41) | 3 | prospective | colon cancer | local recurrence in colon cancer | anastomotic leakage (AL) | 133 | 1990 | OR | random | 2.16 (0.88-5.29) | 0.094 | 0% | SIGN;  level 3 (3 studies) | |
| **Comorbidity** | | | | | | | | | | | | | |  |
| Mills, 2013 (40) | 3 | 2 retrospective;  3 prospective | CRC | overall recurrence in CRC | diabetes | 1678 | 4979 | RR | random | 1.24 (0.99-1.55) | >0.05 | 63.4% | STROBE (16 item); Moderate risk (2 studies: 5-6 items reported); High risk (1 study: 4 items reported) | |
| **Anthropometric indices** | | | | | | | | | | | | | |  |
| Doleman, 2016 (34) | 6 | 5 retrospective;  1 prospective | CRC | overall recurrence in CRC | underweight | 1004+NA | 37223 | RR | random | 1.13 (1.05-1.21) | <0.05 | 0% | STROBE (16 item); Moderate risk (6 studies: 6 items reported) | |
| Doleman, 2016 (34) | 6 | 5 retrospective;  1 prospective | CRC | overall recurrence in CRC | overweight | 1617+NA | 37223 | RR | random | 1.00 (0.96-1.05) | >0.05 | 0% | STROBE (16 item); Moderate risk (6 studies: 6 items reported) | |
| Doleman, 2016 (34) | 6 | 5 retrospective;  1 prospective | CRC | overall recurrence in CRC | obese | 1139+NA | 37223 | RR | random | 1.07 (1.02-1.13) | <0.05 | 0% | STROBE (16 item); Moderate risk (6 studies: 6 items reported) | |

Abbreviation: OR, odds ratio; RR, risk ratio; CI, confidence interval; CRC, colorectal cancer; NOS, Newcastle Ottawa Scale (>=6: low risk, <6: high risk); SIGN, Scottish Intercollegiate Guidelines Network; STROBE, Strengthening the Reporting of Observational studies in Epidemiology; REMARK, Reporting recommendations for tumour Marker prognostic studies; QUIPS, Quality In Prognosis Studies

**Table S8: Criteria for assessing the credibility of the evidence from meta-analyses of observational studies.**

| Category | Criteria |
| --- | --- |
| Convincing | Associations with P < 10^-6^; the largest component study reporting a nominal statistically significant result (P < 0.05); a 95% PI that excluded the null; no large heterogeneity (I² <50%); no evidence of small-study effect (P > 0.10); and no excess significance bias (P > 0.10). |
| Highly suggestive | Associations with P < 10^-6^; and the largest component study reporting a statistically significant result (P < 0.05). |
| Suggestive | Associations with P < 0.001. |
| Weak | Remaining statistically significant associations with P < 0.05. |

| **Table S9: Summary of evidence credibility assessment of 34 unique meta-analyses of observational studies investigating the associations between risk factors and colorectal cancer metastasis** |
| --- |

| **Ref.** | **No.** | **Study design** | **Population** | **Outcome** | **Risk factor** | **n  (event)** | **N (sample)** | **Metric** | **MA model** | **Effect size (95% CI)** | **P-value** | **I²** | **P-egger** | **P-sig** | **95% PI** | **Assessment tool and risk of bias** | **Evidence classification** |  |
| --- | --- | --- | --- | --- | --- | --- | --- | --- | --- | --- | --- | --- | --- | --- | --- | --- | --- | --- |
| **Histopathological risk factor** | | | | | | | | | | | | | | | | | | |
| Choi, 2015 (11) | 5 | retrospective | pT1 CRC | lymph node metastasis in pT1 CRC | vascular invasion | 209 | 1731 | OR | DL | 2.73 (1.98-3.78) | 1.12E-09 | 0% | 0.934 | 0.232 | (1.98-3.78) | No | convincing |  |
| Choi, 2015 (11) | 12 | retrospective | pT1 CRC | lymph node metastasis in pT1 CRC | lymphatic invasion | 392 | 3347 | OR | DL | 6.78 (5.29-8.69) | 1.60E-51 | 0% | 0.080 | 0.467 | (5.29-8.69) | No | highly suggestive |  |
| Cappellesso, 2017 (8) | 41 | 39 retrospective;  2 case-control | pT1 CRC | lymph node metastasis in pT1 CRC | tumour budding | 1240 | 10128 | OR | DL | 6.39 (5.23-7.80) | 6.40E-74 | 29.5% | < 0.0001 | 0.074 | (3.39-12.03) | STROBE; NA | highly suggestive |  |
| Rogers, 2016 (22) | 25 | 24 retrospective;  1 case-control | CRC | lymph node metastasis in CRC | tumour budding | 1808 | 6739 | OR | DL | 4.96 (3.97-6.19) | 4.41E-45 | 53.1% | 0.026 | 0.224 | (2.31-10.63) | NOS (total score: 9); Low risk (24 studies) High risk (1 study) | highly suggestive |  |
| Zhou, 2013 (33) | 7 | retrospective | rectal cancer | lymph node metastasis in rectal cancer | tumour size>1 cm | 140 | 348 | OR | DL | 6.76 (3.25-14.04) | 3.01E-07 | 24.4% | 0.281 | 0.089 | (2.04-22.37) | D.H Checklist (total score: 9); Low risk (7 studies) | highly suggestive |  |
| Beaton, 2013 (6) | 8 | 7 retrospective;  1 prospective | pT1 CRC | lymph node metastasis in pT1 CRC | lymphovascular invasion | 255 | 1695 | OR | DL | 4.81 (3.14-7.36) | 4.62E-13 | 20.7% | 0.657 | 0.000 | (2.42-9.58) | NOS (total score: 9); Low risk (5 studies) High risk (3 studies) | suggestive |  |
| Choi, 2015 (11) | 8 | retrospective | pT1 CRC | lymph node metastasis in pT1 CRC patients who underwent additional surgeries after an endoscopic resection | lymphovascular invasion | 37 | 313 | OR | DL | 5.29 (2.34-11.98) | 6.30E-05 | 0% | 0.784 | 0.138 | (2.34-11.98) | No | suggestive |  |
| Beaton, 2013 (6) | 13 | 12 retrospective;  1 prospective | pT1 CRC | lymph node metastasis in pT1 CRC | poor differentiation | 332 | 2722 | OR | DL | 5.61 (2.90-10.83) | 2.82E-07 | 30.4% | 0.499 | 0.122 | (1.36-23.16) | NOS (total score: 9); Low risk (5 studies) High risk (8 studies) | suggestive |  |
| Zhou, 2013 (33) | 6 | retrospective | rectal cancer | lymph node metastasis in rectal cancer | muscularis properia invasion | 127 | 322 | OR | DL | 5.08 (2.32-11.11) | 4.70E-05 | 26.6% | 0.139 | 0.016 | (1.46-17.73) | D.H Checklist (total score: 9); Low risk (6 studies) | suggestive |  |
| Choi, 2015 (11) | 10 | retrospective | pT1 CRC | lymph node metastasis in pT1 CRC | submucosal invasion ≥ 1mm | 332 | 2922 | OR | DL | 2.95 (1.39-6.27) | 0.005 | 51.7% | 0.314 | 0.000 | (0.50-17.34) | No | weak |  |
| Kang, 2019 (16) | 5 | retrospective | small rectal NETs | lymph node metastasis in small rectal NETs treated by local excision | lymphovascular invasion | 84 | 517 | OR | DL | 5.02 (1.16-21.72) | 0.031 | 72.2% | 0.377 | 0.755 | (0.26-97.93) | NOS (total score: 9);  Moderate risk (4 studies)  High risk (1 study) | weak |  |
| Zhou, 2013 (33) | 2 | retrospective | rectal cancer | lymph node metastasis in rectal cancer | central depression | 33 | 76 | OR | HKSJ | 3.00 (2.10-4.28) | 0.016 | 0% | NA | 0.243 | (2.09-4.29) | D.H Checklist (total score: 9); Low risk (2 studies) | weak |  |
| Siddiqui, 2017 (24) | 3 | retrospective | rectal cancer | synchronous metastasis in rectal cancer | MRI-detected extramural vascular invasion (mrEMVI) | 122 | 804 | OR | HKSJ | 5.65 (2.12-15.05) | 0.017 | 31.6% | 0.764 | 0.737 | (1.19-26.82) | SIGN;  level 3: 3 studies | weak |  |
| Kang, 2019 (16) | 3 | retrospective | small rectal NETs | lymph node metastasis in small rectal NETs treated by local excision | lymphatic invasion | 77 | 493 | OR | HKSJ | 5.54 (0.02-1752.46) | 0.329 | 90.9% | 0.557 | 0.869 | (1.14E-04-2.70E+05) | NOS (total score: 9);  Moderate risk (3 studies) | no association |  |
| Zhou, 2013 (33) | 3 | retrospective | rectal cancer | lymph node metastasis in rectal cancer | vascular invasion | 66 | 168 | OR | HKSJ | 5.86 (0.77-44.62) | 0.064 | 32.9% | 0.628 | 0.024 | (0.28-122.31) | D.H Checklist (total score: 9); Low risk (3 studies) | no association |  |
| Kang, 2019 (16) | 2 | retrospective | small rectal NETs | lymph node metastasis in small rectal NETs treated by local excision | vascular invasion | 62 | 211 | OR | HKSJ | 3.63 (0.05-268.57) | 0.164 | 10.4% | NA | 0.836 | (0.01-2.41E+03) | NOS (total score: 9);  Moderate risk (2 studies) | no association |  |
| Choi, 2015 (11) | 3 | retrospective | pT1 CRC | lymph node metastasis in pT1 CRC patients who underwent additional surgeries after an endoscopic resection | poor or moderate differentiation | 16 | 209 | OR | HKSJ | 3.77 (1.12-123.16) | 0.243 | 35.4% | 0.388 | 0.602 | (0.02-906.34) | No | no association |  |
| **Biomarker** | | | | | | | | | | | | | | | | | | |
| He, 2013 (13) | 14 | retrospective | CRC | lymph node metastasis in CRC | downregulated E-cadherin expression | 658+NA | 1593 | OR | DL | 0.49 (0.34-0.72) | 0.000 | 47.5% | 0.077 | 0.967 | (0.19-1.32) | NOS (total score: 9); Low risk (11 studies) High risk (2 studies) | highly suggestive |  |
| Katsuno, 2008 (17) | 5 | prospective | CRC | hepatic metastasis (distant) in CRC | circulating tumour cells | 38 | 310 | OR | DL | 6.38 (2.67-15.26) | 3.11E-05 | 0% | 0.660 | 0.476 | (2.67-15.26) | No | suggestive |  |
| Li, 2018 (18) | 8 | retrospective | CRC | lymph node metastasis in CRC | low MUC2 expression level | 592 | 1335 | RR | DL | 1.42 (1.19-1.69) | 0.000 | 48.5% | 0.448 | 0.000 | (0.97-2.07) | NOS (total score: 9); Low risk (8 studies) | suggestive |  |
| He, 2013 (13) | 8 | retrospective | CRC | distant metastasis in CRC | downregulated E-cadherin expression | 136+NA | 983 | OR | DL | 0.45 (0.23-0.91) | 0.026 | 76.9% | 0.983 | 0.529 | (0.07-2.83) | NOS (total score: 9); Low risk (6 studies) High risk (2 studies) | weak |  |
| Yang, 2017 (30) | 7 | prospective | CRC | lymph node metastasis in CRC | circulating tumour cells | 721+NA | 1535 | RR | DL | 1.62 (1.17-2.23) | 0.003 | 74.7% | 0.369 | 0.529 | (0.73-3.58) | NOS (total score: 9); Low risk (3 studies) High risk (4 studies) | weak |  |
| Zhou, 2018 (32) | 14 | case-control | CRC | lymph node metastasis in CRC | p16 protein expression | 321+NA | 800+NA | OR | DL | 0.50 (0.30-0.84) | 0.009 | 59.1% | 0.516 | 0.268 | (0.11-2.35) | NOS (total score: 9); Low risk (6 studies) NA risk (8 studies) | weak |  |
| Li, 2014 (19) | 9 | 6 prospective; 3 case-control | CRC | distant metastasis in CRC | cyclin D1 overexpression | 88+NA | 1515 | OR | DL | 0.60 (0.36-0.99) | 0.047 | 64.8% | 0.315 | 0.000 | (0.17-2.11) | NOS (total score: 9); Low risk (9 studies) | weak |  |
| Chen, 2013 (10) | 5 | retrospective | CRC | distant metastasis in CRC | ℬ-catenin overexpression in the nucleus | 217 | 628 | OR | DL | 0.48 (0.29-0.79) | 0.004 | 60.2% | 0.013 | 0.147 | (0.19-1.23) | NOS (total score: 9); Low risk (4 studies) High risk (1 study) | weak |  |
| Huang, 2016 (14) | 3 | retrospective | CRC | lymph node metastasis in CRC | CD147 expression | 374 | 815 | OR | HKSJ | 1.41 (0.39-5.01) | 0.367 | 71% | 0.475 | 0.000 | (0.15-13.06) | ELCWP scale; NA | no association |  |
| Huang, 2016 (14) | 2 | retrospective | CRC | distant metastasis in CRC | CD147 expression | 56 | 538 | OR | HKSJ | 2.32 (1.34E-06 -4.03E+06) | 0.592 | 85.3% | NA | 0.000 | (2.26E-10-2.39E+10) | ELCWP scale; NA | no association |  |
| Wang, 2012 (27) | 7 | retrospective | CRC | lymph node metastasis in CRC | CD133 expression | 751 | 1629 | OR | DL | 1.15 (0.82-1.62) | 0.414 | 18.5% | 0.069 | 0.452 | (0.69-1.93) | No | no association |  |
| Chen, 2013 (9) | 4 | retrospective | CRC | distant metastasis in CRC | CD133 expression | 95 | 1064 | RR | HKSJ | 1.54 (0.39-6.09) | 0.394 | 70.9% | 0.282 | 0.120 | (0.09-25.23) | No | no association |  |
| Wu, 2015 (29) | 8 | retrospective | CRC | lymph node metastasis in CRC | HER-2 immunohistochemical expression | 369 | 1289 | OR | DL | 1.90 (0.90-4.02) | 0.093 | 82.3% | 0.335 | 0.340 | (0.27-13.57) | No | no association |  |
| **Genetic risk factor** | | | | | | | | | | | | | | | | | | |
| Li, 2017 (20) | 4 | retrospective | CRC | lymph node metastasis in CRC | BRAF mutation | 100 | 1142 | OR | HKSJ | 0.75 (0.49-1.14) | 0.117 | 9.2% | 0.552 | 0.412 | (0.38-1.48) | No | no association |  |
| Wang, 2014 (26) | 3 | retrospective | CRC | lymph node metastasis in CRC | RASSF1A promoter methylation | 91 | 184 | OR | HKSJ | 1.61 (0.16-16.16) | 0.468 | 57.9% | 0.808 | 0.098 | (0.04-73.35) | NOS (total score: 9);  Low risk (2 studies)  Moderate risk (1 study) | no association |  |
| Wang, 2014 (26) | 4 | retrospective | CRC | distant metastasis in CRC | RASSF1A promoter methylation | 173 | 417 | OR | HKSJ | 2.57 (0.64-10.24) | 0.119 | 51.1% | 0.667 | 0.835 | (0.20-32.41) | NOS (total score: 9);  Low risk (3 studies)  Moderate risk (1 study) | no association |  |
| **Demographic risk factor** | | | | | | | | | | | | | | | | | | |
| Ichimasa, 2017 (15) | 4 | retrospective | pT1 CRC | lymph node metastasis in pT1 CRC | female gender | 113 | 1329 | RR | HKSJ | 2.23 (0.78-6.42) | 0.094 | 92.1% | 0.175 | 0.263 | (0.25-20.27) | GRADE;  Low (+OOO) risk (4 studies) | no association |  |

Abbreviation: OR, odds ratio; RR, risk ratio; CI, confidence interval; P-egger, P value for Egger’s test; P-sig, P value for excess significance bias; PI, prediction interval; No., number of included studies; NA, not available (where the number of expected significant results was greater than the number observed); CRC, colorectal cancer; NET, neuroendocrine tumour; MINORS, Methodological index for non-randomized studies; NOS, Newcastle Ottawa Scale (>=6: low risk, <6: high risk); D.H Checklist, Duckitt and Harrington Checklist; ELCWP scale, European Lung Cancer Working Party; GRADE, The Grading of Recommendations Assessment, Development and Evaluation; SIGN, Scottish Intercollegiate Guidelines Network; STROBE, Strengthening the Reporting of Observational studies in Epidemiology

**Table S10: Summary of evidence credibility assessment of 17 unique meta-analyses of observational studies investigating the associations between risk factors and colorectal cancer recurrence**

| **Ref.** | **No.** | **Study design** | **Population** | **Outcome** | **Risk factor** | | **n  (event)** | **N (sample)** | **Metric** | **MA model** | **Effect size (95% CI)** | **P-value** | **I²** | **P-egger** | **P-sig** | **95% PI** | **Assessment tool and risk of bias** | **Evidence classification** |
| --- | --- | --- | --- | --- | --- | --- | --- | --- | --- | --- | --- | --- | --- | --- | --- | --- | --- | --- |
| **Histopathological risk factor** | | | | | | | | | | | | | | | | | | |
| Knijn, 2016 (36) | 5 | retrospective | rectal cancer | local recurrence in rectal cancer | | perineural invasion (PNI) | 146 | 1700 | RR | DL | 3.21 (2.33-4.44) | 1.32E-12 | 21.7% | 0.059 | 0.155 | (2.02-5.12) | REMARK  Low risk (2 studies: 83.3% items reported); Moderate risk (3 studies:61.1%, 50% items reported) | highly suggestive |
| Rogers, 2016 (22) | 12 | 11 retrospective;  1 case-control | CRC | overall recurrence in CRC | | tumour budding | 551 | 2773 | OR | DL | 5.50 (3.65-8.29) | 4.23E-16 | 61% | 0.677 | 0.000 | (1.77-17.07) | NOS (total score: 9); Low risk (12 studies) | highly suggestive |
| Veronese, 2015 (43) | 9 | prospective | CRC | overall recurrence in CRC | | extranodal extension (ENE) | 389 | 877 | RR | DL | 2.07 (1.65-2.61) | 5.48E-10 | 47.2% | 0.003 | 0.163 | (1.24-3.46) | NOS (total score: 9); Low risk (9 studies) | highly suggestive |
| Siddiqui, 2017 (24) | 6 | retrospective | rectal cancer | distant metastatic recurrence in rectal cancer | | MRI-detected extramural vascular invasion (mrEMVI) | 284 | 1262 | OR | DL | 3.91 (2.61-5.86) | 3.35E-11 | 41.4% | 0.736 | 0.000 | (1.86-8.22) | SIGN; level 3 (6 studies) | highly suggestive |
| Knijn, 2018 (37) | 2 | 1 prospective;  1 case-control | CRC | local recurrence in CRC | | intramural vascular invasion (IMVI) | 96 | 503 | RR | HKSJ | 1.55 (0.11-21.28) | 0.281 | 7.9% | NA | 0.451 | (0.03-87.47) | REMARK  Low risk (1 study: 88.9% items reported); Moderate risk (1 study:55% items reported) | no association |
| **Biomarker** | | | | | | | | | | | | | | | | | | |
| Rekhraj, 2008 (42) | 6 | 5 retrospective;  1 prospective | CRC | overall recurrence in CRC | | absence of peritoneal free tumour cells in pre-resection | 164 | 593 | OR | DL | 0.38 (0.16-0.91) | 0.029 | 45.7% | 0.260 | 0.001 | (0.07-1.96) | No | weak |
| Rekhraj, 2008 (42) | 3 | 2 retrospective;  1 prospective | CRC | overall recurrence in CRC | | absence of peritoneal free tumour cells in post-resection | 57 | 252 | OR | HKSJ | 0.07 (0.02-0.21) | 0.009 | 3.3% | 0.743 | 0.000 | (0.02-0.26) | No | weak |
| **Genetic risk factor** | | | | | | | | | | | | | | | | | | |
| Kunzmann, 2013 (38) | 8 | retrospective | CRC | overall recurrence in CRC | | PTGS2 (also known as COX-2) | 232+NA | 1516 | HR | DL | 2.78 (1.76-4.40) | 1.22E-05 | 36.2% | 0.770 | 0.169 | (1.15-6.75) | No | suggestive |
| **Clinical risk factor** | | | | | | | | | | | | | | | | | | |
| Ha, 2017 (35) | 26 | 12 retrospective;  14 prospective | CRC | local recurrence in CRC | | anastomotic leakage (AL) | 3675 | 39745 | RR | DL | 1.90 (1.48-2.43) | 3.35E-07 | 77.7% | 0.017 | 0.000 | (0.69-5.23) | QUIPS;  NA | suggestive |
| Lu, 2016 (39) | 11 | 6 retrospective;  5 prospective | rectal cancer | local recurrence in rectal cancer | | anastomotic leakage (AL) | 1199 | 13665 | OR | DL | 1.61 (1.25-2.08) | 2.65E-04 | 36.9% | 0.143 | 0.001 | (0.92-2.82) | NOS (total score: 9);  Low risk (11 studies) | suggestive |
| Ha, 2017 (35) | 11 | 5 retrospective;  6 prospective | CRC | distant recurrence in CRC | | anastomotic leakage (AL) | 2086 | 10392 | RR | DL | 1.20 (0.94-1.52) | 0.136 | 59.4% | 0.245 | 0.226 | (0.66-2.18) | QUIPS;  NA | no association |
| Lu, 2016 (39) | 4 | 1 retrospective;  3 prospective | rectal cancer | distant recurrence in rectal cancer | | anastomotic leakage (AL) | 1163 | 5221 | OR | HKSJ | 1.06 (0.72-1.58) | 0.657 | 39.3% | 0.991 | 0.421 | (0.51-2.22) | NOS (total score: 9); Low risk (4 studies) | no association |
| Mirnezami, 2011 (41) | 3 | prospective | colon cancer | local recurrence in colon cancer | | anastomotic leakage (AL) | 133 | 1990 | OR | HKSJ | 2.19 (0.55-8.68) | 0.135 | 7.9% | 0.662 | 0.358 | (0.38-12.61) | SIGN;  level 3 (3 studies) | no association |
| **Comorbidity** | | | | | | | | | | | | | | | | | | |
| Mills, 2013 (40) | 3 | 2 retrospective;  3 prospective | CRC | overall recurrence in CRC | | diabetes | 1678 | 4979 | RR | HKSJ | 1.26 (0.70-2.30) | 0.233 | 76.3% | 0.184 | 0.417 | (0.43-3.71) | STROBE (16 item); Moderate risk (2 studies: 5-6 items reported); High risk (1 study: 4 items reported) | no association |
| **Anthropometric indices** | | | | | | | | | | | | | | | | | | |
| Doleman, 2016 (34) | 6 | 5 retrospective;  1 prospective | CRC | overall recurrence in CRC | | underweight | 1004+NA | 37223 | RR | DL | 1.13 (1.05-1.21) | 0.002 | 0% | 0.799 | 0.281 | (1.05-1.21) | STROBE (16 item); Moderate risk (6 studies: 6 items reported) | weak |
| Doleman, 2016 (34) | 6 | 5 retrospective;  1 prospective | CRC | overall recurrence in CRC | | obese | 1139+NA | 37223 | RR | DL | 1.07 (1.02-1.13) | 0.007 | 0% | 0.387 | 0.038 | (1.02-1.13) | STROBE (16 item); Moderate risk (6 studies: 6 items reported) | weak |
| Doleman, 2016 (34) | 6 | 5 retrospective;  1 prospective | CRC | overall recurrence in CRC | | overweight | 1617+NA | 37223 | RR | DL | 1.00 (0.96-1.05) | 0.887 | 0% | 0.593 | 0.039 | (0.96-1.05) | STROBE (16 item); Moderate risk (6 studies: 6 items reported) | no association |

Abbreviation: OR, odds ratio; RR, risk ratio; CI, confidence interval; P-egger, P value for Egger’s test; P-sig, P value for excess significance bias; PI, prediction interval; No., number of included studies; NA, not available (where the number of expected significant results was greater than the number observed); CRC, colorectal cancer; NOS, Newcastle Ottawa Scale (>=6: low risk, <6: high risk); SIGN, Scottish Intercollegiate Guidelines Network; STROBE, Strengthening the Reporting of Observational studies in Epidemiology; REMARK, Reporting recommendations for tumour Marker prognostic studies; QUIPS, Quality In Prognosis Studies

| **Table S11: Sensitivity analysis of 16 unique meta-analyses of observational studies investigating the associations between risk factors and colorectal cancer metastasis (at presentation) and evidence credibility assessment** |
| --- |

| **Ref.** | **No.** | **Study design** | **Population** | **Outcome** | **Risk factor** | **n  (event)** | **N (sample)** | **Metric** | **MA model** | **Effect size (95% CI)** | **P-value** | **I²** | **P-egger** | **P-sig** | **95% PI** | **Assessment tool and risk of bias** | **Evidence classification** |
| --- | --- | --- | --- | --- | --- | --- | --- | --- | --- | --- | --- | --- | --- | --- | --- | --- | --- |
|  | | **Histopathological risk factor** | | | | | | | | | | | | | | | |
| Cappellesso, 2017 (8) | 14 | retrospective | pT1 CRC | lymph node metastasis at presentation in pT1 CRC | tumour budding | 345 | 2610 | OR | DL | 5.37 (4.05-7.11) | 1.53E-31 | 7.9% | 0.069 | 0.398 | (3.57-8.08) | STROBE; NA | highly suggestive |
| Rogers, 2016 (22) | 11 | retrospective | CRC | lymph node metastasis at presentation in CRC | tumour budding | 421 | 2617 | OR | DL | 4.74 (3.33-6.74) | 5.73E-18 | 41.3% | 0.016 | 0.023 | (2.14-10.47) | NOS (total score: 9);  Low risk (11 studies) | highly suggestive |
| Zhou, 2013 (33) | 5 | retrospective | rectal cancer | lymph node metastasis at presentation in rectal cancer | tumour size>1 cm | 101 | 242 | OR | DL | 8.73 (4.29-17.78) | 2.34E-09 | 0% | 0.676 | 0.353 | (4.29-17.78) | D.H Checklist (total score: 9);  Low risk (5 studies) | highly suggestive |
| Choi, 2015 (11) | 3 | retrospective | pT1 CRC | lymph node metastasis at presentation in pT1 CRC | lymphatic invasion | 86 | 654 | OR | HKSJ | 5.68 (4.06-7.94) | 0.002 | 0.6% | 0.415 | 0.797 | (3.93-8.21) | No | weak |
| Kang, 2019 (16) | 5 | retrospective | small rectal NETs | lymph node metastasis at presentation in small rectal NETs treated by local excision | lymphovascular invasion | 84 | 517 | OR | DL | 5.02 (1.16-21.72) | 0.031 | 72.2% | 0.377 | 0.755 | (0.26-97.93) | NOS (total score: 9);  Moderate risk (4 studies)  High risk (1 study) | weak |
| Zhou, 2013 (33) | 4 | retrospective | rectal cancer | lymph node metastasis at presentation in rectal cancer | muscularis properia invasion | 86 | 209 | OR | HKSJ | 6.52 (1.52-27.99) | 0.026 | 31.6% | 0.515 | 0.038 | (0.54-78.33) | D.H Checklist (total score: 9);  Low risk (4 studies) | weak |
| Siddiqui, 2017 (24) | 3 | retrospective | rectal cancer | synchronous metastasis at presentation in rectal cancer | MRI-detected extramural vascular invasion (mrEMVI) | 122 | 804 | OR | HKSJ | 5.65 (2.12-15.05) | 0.017 | 31.6% | 0.764 | 0.737 | (1.19-26.82) | SIGN;  level 3: 3 studies | weak |
| Choi, 2015 (11) | 3 | retrospective | pT1 CRC | lymph node metastasis at presentation in pT1 CRC | submucosal invasion ≥ 1mm | 66 | 564 | OR | HKSJ | 2.60 (0.15-43.70) | 0.283 | 33.1% | 0.246 | 0.000 | (0.04-191.27) | No | no association |
| Kang, 2019 (16) | 3 | retrospective | small rectal NETs | lymph node metastasis at presentation in small rectal NETs treated by local excision | lymphatic invasion | 77 | 493 | OR | HKSJ | 5.54 (0.02-1752.46) | 0.329 | 90.9% | 0.557 | 0.869 | (1.14E-04-2.70E+05) | NOS (total score: 9);  Moderate risk (3 studies) | no association |
| Choi, 2015 (11) | 2 | retrospective | pT1 CRC | lymph node metastasis at presentation in pT1 CRC | vascular invasion | 65 | 477 | OR | HKSJ | 2.68 (0.27-27.00) | 0.116 | 4.0% | NA | 0.215 | (0.19-38.61) | No | no association |
| Zhou, 2013 (33) | 3 | retrospective | rectal cancer | lymph node metastasis at presentation in rectal cancer | vascular invasion | 66 | 168 | OR | HKSJ | 5.86 (0.77-44.62) | 0.064 | 32.9% | 0.628 | 0.024 | (0.28-122.31) | D.H Checklist (total score: 9); Low risk (3 studies) | no association |
| Kang, 2019 (16) | 2 | retrospective | small rectal NETs | lymph node metastasis at presentation in small rectal NETs treated by local excision | vascular invasion | 62 | 211 | OR | HKSJ | 3.63 (0.05-268.57) | 0.164 | 10.4% | NA | 0.836 | (0.01-2.41E+03) | NOS (total score: 9);  Moderate risk (2 studies) | no association |
| Choi, 2015 (11) | 2 | retrospective | pT1 CRC | lymph node metastasis at presentation in pT1 CRC patients who underwent additional surgeries after an endoscopic resection | lymphovascular invasion | 12 | 92 | OR | HKSJ | 5.29 (0.16-171.94) | 0.104 | 0.6% | NA | 0.264 | (0.14-207.14) | No | no association |
| Beaton, 2013 (6) | 2 | retrospective | pT1 CRC | lymph node metastasis at presentation in pT1 CRC | poor differentiation | 10 | 98 | OR | HKSJ | 2.65 (0.01-598.02) | 0.263 | 1.4% | NA | 0.328 | (0.01-931.84) | NOS (total score: 9);  Low risk (1 study)  High risk (1 study) | no association |
| **Biomarker** | | | | | | | | | | | | | | | | | |
| He, 2013 (13) | 2 | retrospective | CRC | lymph node metastasis at presentation in CRC | downregulated E-cadherin expression | 109 | 230 | OR | HKSJ | 0.36 (0.02-7.52) | 0.145 | 5.3% | NA | 0.020 | (0.01-13.33) | NOS (total score: 9); Low risk (2 studies) | no association |
| Wang, 2012 (27) | 3 | retrospective | CRC | lymph node metastasis at presentation in CRC | CD133 expression | 225 | 465 | OR | HKSJ | 1.49 (0.87-2.57) | 0.086 | 3.1% | 0.989 | 0.171 | (0.79-2.83) | No | no association |

Abbreviation: OR, odds ratio; RR, risk ratio; CI, confidence interval; P-egger, P value for Egger’s test; P-sig, P value for excess significance bias; PI, prediction interval; No., number of included studies; NA, not available (where the number of expected significant results was greater than the number observed); CRC, colorectal cancer; NET, neuroendocrine tumour; MINORS, Methodological index for non-randomized studies; NOS, Newcastle Ottawa Scale (>=6: low risk, <6: high risk); D.H Checklist, Duckitt and Harrington Checklist; ELCWP scale, European Lung Cancer Working Party; GRADE, The Grading of Recommendations Assessment, Development and Evaluation; SIGN, Scottish Intercollegiate Guidelines Network; STROBE, Strengthening the Reporting of Observational studies in Epidemiology

**Table S12: Sensitivity analysis of 13 unique meta-analyses of observational studies investigating the associations between risk factors and colorectal cancer recurrence (local/ distant) and evidence credibility assessment**

| **Ref.** | **No.** | **Study design** | **Population** | **Outcome** | **Risk factor** | **n  (event)** | **N (sample)** | **Metric** | **MA model** | **Effect size (95% CI)** | **P-value** | **I²** | **P-egger** | **P-sig** | **95% PI** | **Assessment tool and risk of bias** | **Evidence classification** |  |
| --- | --- | --- | --- | --- | --- | --- | --- | --- | --- | --- | --- | --- | --- | --- | --- | --- | --- | --- |
| **Histopathological risk factor** | | | | | | | | | | | | | | | | | | |
| Knijn, 2016 (36) | 5 | retrospective | rectal cancer | local recurrence in rectal cancer | perineural invasion (PNI) | 146 | 1700 | RR | DL | 3.21 (2.33-4.44) | 1.32E-12 | 21.7% | 0.059 | 0.155 | (2.02-5.12) | REMARK  Low risk (2 studies: 83.3% items reported); Moderate risk (3 studies:61.1%, 50% items reported) | highly suggestive |  |
| Siddiqui, 2017 (24) | 6 | retrospective | rectal cancer | distant metastatic recurrence in rectal cancer | MRI-detected extramural vascular invasion (mrEMVI) | 284 | 1262 | OR | DL | 3.91 (2.61-5.86) | 3.35E-11 | 41.4% | 0.736 | 0.000 | (1.86-8.22) | SIGN; level 3 (6 studies) | highly suggestive |  |
| Knijn, 2018 (37) | 2 | 1 prospective;  1 case-control | CRC | local recurrence in CRC | intramural vascular invasion (IMVI) | 96 | 503 | RR | HKSJ | 1.55 (0.11-21.28) | 0.281 | 7.9% | NA | 0.451 | (0.03-87.47) | REMARK  Low risk (1 study: 88.9% items reported); Moderate risk (1 study:55% items reported) | no association |  |
| **Biomarker** | | | | | | | | | | | | | | | | | | |
| Katsuno, 2008 (17) | 4 | prospective | CRC | *hepatic metastasis (distant) after being disease free in CRC | circulating tumour cells | 36 | 267 | OR | HKSJ | 6.05 (4.64-7.89) | 2.17E-04 | 0.1% | 0.138 | 0.582 | (4.54-8.06) | No | suggestive |  |
| He, 2013 (13) | 3 | retrospective | CRC | *distant metastasis after being disease free in CRC | downregulated E-cadherin expression | 44+NA | 498 | OR | HKSJ | 0.28 (0.05-1.71) | 0.094 | 67.6% | 0.493 | 0.002 | (0.01-6.28) | NOS (total score: 9); Low risk (2 studies) High risk (1 study) | no association |  |
| Chen, 2013 (9) | 2 | retrospective | CRC | *distant metastasis after being disease free in CRC | CD133 expression | 63 | 340 | RR | HKSJ | 1.37 (0.06-31.30) | 0.419 | 21.8% | NA | 0.709 | (0.02-77.88) | No | no association |  |
| Li, 2014 (19) | 2 | prospective | CRC | *distant metastasis after being disease free in CRC | cyclin D1 overexpression | 18 | 317 | OR | HKSJ | 1.53 (4.56E-06-5.10E+05) | 0.746 | 87.3% | NA | 2.93E-03 | (1.73E-09-1.34E+09) | NOS (total score: 9); Low risk (2 studies) | no association |  |
| Chen, 2013 (10) | 2 | retrospective | CRC | *distant metastasis after being disease free in CRC | ℬ-catenin overexpression in the nucleus | 115 | 251 | OR | HKSJ | 0.55 (2.95E-03-101.66) | 0.381 | 53.4% | NA | 0.797 | (2.72E-04-1.10E+03) | NOS (total score: 9); Low risk (2 studies) | no association |  |
| **Clinical risk factor** | | | | | | | | | | | | | | | | | | |
| Ha, 2017 (35) | 26 | 12 retrospective;  14 prospective | CRC | local recurrence in CRC | anastomotic leakage (AL) | 3675 | 39745 | RR | DL | 1.90 (1.48-2.43) | 3.35E-07 | 77.7% | 0.017 | 0.000 | (0.69-5.23) | QUIPS;  NA | suggestive |  |
| Lu, 2016 (39) | 11 | 6 retrospective;  5 prospective | rectal cancer | local recurrence in rectal cancer | anastomotic leakage (AL) | 1199 | 13665 | OR | DL | 1.61 (1.25-2.08) | 2.65E-04 | 36.9% | 0.143 | 0.001 | (0.92-2.82) | NOS (total score: 9);  Low risk (11 studies) | suggestive |  |
| Ha, 2017 (35) | 11 | 5 retrospective;  6 prospective | CRC | distant recurrence in CRC | anastomotic leakage (AL) | 2086 | 10392 | RR | DL | 1.20 (0.94-1.52) | 0.136 | 59.4% | 0.245 | 0.226 | (0.66-2.18) | QUIPS;  NA | no association |  |
| Lu, 2016 (39) | 4 | 1 retrospective;  3 prospective | rectal cancer | distant recurrence in rectal cancer | anastomotic leakage (AL) | 1163 | 5221 | OR | HKSJ | 1.06 (0.72-1.58) | 0.657 | 39.3% | 0.991 | 0.421 | (0.51-2.22) | NOS (total score: 9); Low risk (4 studies) | no association |  |
| Mirnezami, 2011 (41) | 3 | prospective | colon cancer | local recurrence in colon cancer | anastomotic leakage (AL) | 133 | 1990 | OR | HKSJ | 2.19 (0.55-8.68) | 0.135 | 7.9% | 0.662 | 0.358 | (0.38-12.61) | SIGN;  level 3 (3 studies) | no association |  |

*distant metastasis after being disease free in CRC, can be classified as distant metastatic recurrence in CRC (the original meta-analysis contains the remaining individual studies did not provide when metastasis was assessed, thus, cannot be included in the sensitivity analysis)

Abbreviation: OR, odds ratio; RR, risk ratio; CI, confidence interval; P-egger, P value for Egger’s test; P-sig, P value for excess significance bias; PI, prediction interval; No., number of included studies; NA, not available (where the number of expected significant results was greater than the number observed); CRC, colorectal cancer; NOS, Newcastle Ottawa Scale (>=6: low risk, <6: high risk); SIGN, Scottish Intercollegiate Guidelines Network; STROBE, Strengthening the Reporting of Observational studies in Epidemiology; REMARK, Reporting recommendations for tumour Marker prognostic studies; QUIPS, Quality In Prognosis Studies

**Table S13: Quality and risk of bias assessment (AMSTAR 2.0) for the evidence represented at least 3-fold changes in the odds of the outcome**

|  |  | | **AMSTAR Items** | | | | | | | | | | | | | | | | |
| --- | --- | --- | --- | --- | --- | --- | --- | --- | --- | --- | --- | --- | --- | --- | --- | --- | --- | --- | --- |
| **References** | **Risk factor** | **Outcome** | **1** | **2*** | **3** | **4*** | **5** | **6** | **7*** | **8** | **9*** | **10** | **11*** | **12** | **13*** | **14** | **15*** | **16** | **Rating** |
| **Risk factors and CRC metastasis** | | | | | | | | | | | | | | | | | | | |
| Choi, 2015 (11) | lymphatic invasion | lymph node metastasis in pT1 CRC | Yes | No | No | PY | Yes | No | No | No | No | No | NRSI  Yes | No | No | Yes | Yes | Yes | CL |
|  | lymphovascular invasion | lymph node metastasis in pT1 CRC patients who underwent additional surgeries after an endoscopic resection |  |  |  |  |  |  |  |  |  |  |  |  |  |  |  |  |  |
| Cappellesso, 2017 (8) | tumour budding | lymph node metastasis in pT1 CRC | Yes | No | No | PY | Yes | Yes | No | No | NRSI  PY | No | NRSI  Yes | No | No | Yes | Yes | Yes | CL |
| Rogers, 2016 (22) | tumour budding | lymph node metastasis in CRC | Yes | No | Yes | PY | Yes | Yes | No | No | NRSI  PY | No | NRSI  Yes | No | No | No | Yes | Yes | CL |
| Beaton, 2013 (6) | lymphovascular invasion | lymph node metastasis in pT1 CRC | Yes | No | No | PY | Yes | Yes | No | No | NRSI  PY | No | NRSI  Yes | No | Yes | No | No | No | CL |
|  | poor differentiation | lymph node metastasis in pT1 CRC |  |  |  |  |  |  |  |  |  |  |  |  |  |  |  |  |  |
| Zhou, 2013 (33) | tumour size >1 cm | lymph node metastasis in rectal cancer | Yes | No | Yes | PY | Yes | Yes | No | No | NRSI  PY | No | NRSI  Yes | No | No | Yes | No | No | CL |
|  | muscularis properia invasion | lymph node metastasis in rectal cancer |  |  |  |  |  |  |  |  |  |  |  |  |  |  |  |  |  |
|  | central depression | lymph node metastasis in rectal cancer |  |  |  |  |  |  |  |  |  |  |  |  |  |  |  |  |  |
| Katsuno, 2008 (17) | circulating tumour cells | hepatic metastasis (distant) in CRC | Yes | No | No | PY | Yes | Yes | No | No | No | No | NRSI  Yes | No | No | No | No | No | CL |
| Kang, 2019 (16) | lymphovascular invasion | lymph node metastasis in small rectal NETs treated by local excision | Yes | No | No | PY | Yes | Yes | No | No | NRSI  PY | No | NRSI  Yes | No | Yes | No | Yes | No | CL |
| Siddiqui, 2017 (24) | MRI-detected extramural vascular invasion (mrEMVI) | synchronous metastasis in rectal cancer | Yes | Yes | No | PY | Yes | Yes | No | PY | NRSI  PY | No | NRSI  Yes | No | No | Yes | No | Yes | CL |
| **Risk factors and CRC recurrence** | | | | | | | | | | | | | | | | | | | |
| Rogers, 2016 (22) | tumour budding | overall recurrence in CRC | Yes | No | Yes | PY | Yes | Yes | No | No | NRSI  PY | No | NRSI  Yes | No | No | No | Yes | Yes | CL |
| Knijn, 2016 (36) | perineural invasion (PNI) | local recurrence in rectal cancer | No | No | No | No | Yes | Yes | No | PY | NRSI  PY | No | NRSI  Yes | No | No | Yes | Yes | Yes | CL |
| Siddiqui, 2017 (24) | MRI-detected extramural vascular invasion (mrEMVI) | distant metastatic recurrence in rectal cancer | Yes | Yes | No | PY | Yes | Yes | No | PY | NRSI  PY | No | NRSI  Yes | No | No | Yes | No | Yes | CL |
| Rekhraj, 2008 (42) | absence of peritoneal free tumour cells in post-resection | overall recurrence in CRC | Yes | No | No | PY | Yes | Yes | No | No | No | No | NRSI  Yes | No | No | Yes | Yes | No | CL |

* critical domains.

PY=partial yes; CL=critically low; NRSI= non-randomized studies

**AMSTAR Checklist 2:** Shea, B.J., Reeves, B.C., Wells, G., Thuku, M., Hamel, C., Moran, J., Moher, D., Tugwell, P., Welch, V., Kristjansson, E. and Henry, D.A., 2017. AMSTAR 2: a critical appraisal tool for systematic reviews that include randomised or non-randomised studies of healthcare interventions, or both*. Bmj, 358*, p.j4008.

1. Did the research questions and inclusion criteria for the review include the components of PICO?

The research questions and inclusion criteria for the review should include Population, Intervention, Comparator group, and Outcome.

Note: Timeframe for follow-up is optional (recommended) to get a yes.

2. Did the report of the review contain an explicit statement that the review methods were established prior to the conduct of the review and did the report justify any significant deviations from the protocol?

For Partial Yes:

The authors state that they had a written protocol or guide that included ALL the following: review question(s), a search strategy, inclusion/exclusion criteria, a risk of bias assessment.

For Yes:

As for partial yes, plus the protocol should be registered and should also have specified: a meta-analysis/synthesis plan, if appropriate, and a plan for investigating causes of heterogeneity, justification for any deviations from the protocol.

3. Did the review authors explain their selection of the study designs for inclusion in the review?

For Yes, the review should satisfy ONE of the following: explanation for including only RCTs, OR explanation for including only NRSI, OR explanation for including both RCTs and NRSI.

4. Did the review authors use a comprehensive literature search strategy?

For Partial Yes (ALL the following): searched at least 2 databases (relevant to research question), provided key word and/or search strategy, justified publication restrictions (eg, language). For Yes, should also have (all the following): searched the reference lists/bibliographies of included studies, searched trial/study registries, included/consulted content experts in the field, where relevant, searched for grey literature, conducted search within 24 months of completion of the review.

5. Did the review authors perform study selection in duplicate?

For Yes, either ONE of the following: at least two reviewers independently agreed on selection of eligible studies and achieved consensus on which studies to include, OR two reviewers selected a sample of eligible studies and achieved good agreement (at least 80 per cent), with the remainder selected by one reviewer.

6. Did the review authors perform data extraction in duplicate?

For Yes, either ONE of the following: at least two reviewers achieved consensus on which data to extract from included studies, OR two reviewers extracted data from a sample of eligible studies and achieved good agreement (at least 80 per cent), with the remainder extracted by one reviewer.

7. Did the review authors provide a list of excluded studies and justify the exclusions?

For Partial Yes: provided a list of all potentially relevant studies that were read in full text form but excluded from the review

For Yes, must also have: justified the exclusion from the review of each potentially relevant study.

8. Did the review authors describe the included studies in adequate detail?

For Partial Yes (ALL the following): described populations, described interventions, described comparators, described outcomes, described research designs.

For Yes, should also have ALL the following: described population in detail, described intervention and comparator in detail (including doses where relevant), described study’s setting, timeframe for follow-up.

9. Did the review authors use a satisfactory technique for assessing the risk of bias (RoB) in individual studies that were included in the review?

RCTs

For Partial Yes, must have assessed RoB from unconcealed allocation, and lack of blinding of patients and assessors when assessing outcomes (unnecessary for objective outcomes such as all-cause mortality).

For Yes, must also have assessed RoB from: allocation sequence that was not truly random, and selection of the reported result from among multiple measurements or analyses of a specified outcome.

NRSI

For Partial Yes, must have assessed RoB: from confounding, and from selection bias.

For Yes, must also have assessed RoB: methods used to ascertain exposures and outcomes, and selection of the reported result from among multiple measurements or analyses of a specified outcome.

10. Did the review authors report on the sources of funding for the studies included in the review?

For Yes, must have reported on the sources of funding for individual studies included in the review. Note: Reporting that the reviewers looked for this information but it was not reported by study authors also qualifies.

11. If meta-analysis was performed did the review authors use appropriate methods for statistical combination of results?

RCTs

For Yes: the authors justified combining the data in a meta-analysis, AND they used an appropriate weighted technique to combine study results and adjusted for heterogeneity if present, AND investigated the causes of any heterogeneity.

NRSI

For Yes: the authors justified combining the data in a meta-analysis, AND they used an appropriate weighted technique to combine study results, adjusting for heterogeneity if present, AND they statistically combined effect estimates from NRSI that were adjusted for confounding, rather than combining raw data, or justified combining raw data when adjusted effect estimates were not available, AND they reported separate summary estimates for RCTs and NRSI separately when both were included in the review.

12. If meta-analysis was performed, did the review authors assess the potential impact of RoB in individual studies on the results of the meta-analysis or other evidence synthesis?

For Yes: included only low risk of bias RCTs, OR, if the pooled estimate was based on RCTs and/or NRSI at variable RoB, the authors performed analyses to investigate possible impact of RoB on summary estimates of effect.

13. Did the review authors account for RoB in individual studies when interpreting/discussing the results of the review?

For Yes: included only low risk of bias RCTs, OR, if RCTs with moderate or high RoB, or NRSI were included the review provided a discussion of the likely impact of RoB on the results.

14. Did the review authors provide a satisfactory explanation for, and discussion of, any heterogeneity observed in the results of the review?

For Yes: there was no significant heterogeneity in the results, OR if heterogeneity was present the authors performed an investigation of sources of any heterogeneity in the results and discussed the impact of this on the results of the review.

15. If they performed quantitative synthesis did the review authors carry out an adequate investigation of publication bias (small study bias) and discuss its likely impact on the results of the review?

For Yes: performed graphical or statistical tests for publication bias and discussed the likelihood and magnitude of impact of publication bias.

16. Did the review authors report any potential sources of conflict of interest, including any funding they received for conducting the review?

For Yes: the authors reported no competing interests, OR the authors described their funding sources and how they managed potential conflicts of interest.

**Rating overall confidence in the results of the review**

High: No or one non-critical weakness: the systematic review provides an accurate and comprehensive summary of the results of the available studies that address the question of interest.

Moderate: More than one non-critical weakness*: the systematic review has more than one weakness but no critical flaws. It may provide an accurate summary of the results of the available studies that were included in the review.

Low: One critical flaw with or without non-critical weaknesses: the review has a critical flaw and may not provide an accurate and comprehensive summary of the available studies that address the question of interest.

Critically low: More than one critical flaw with or without non-critical weaknesses: the review has more than one critical flaw and should not be relied on to provide an accurate and comprehensive summary of the available studies.

*Multiple non-critical weaknesses may diminish confidence in the review and it may be appropriate to move the overall appraisal down from moderate to low confidence.
